# Activity-based chemoproteomic profiling reveals the active kinome of *Leishmania*

**DOI:** 10.3389/fphar.2025.1687590

**Published:** 2026-01-06

**Authors:** Exequiel O. J. Porta, Karunakaran Kalesh, Patrick G. Steel

**Affiliations:** 1 Department of Chemistry, Durham University, Durham, United Kingdom; 2 School of Health and Life Sciences, Teesside University, Middlesbrough, United Kingdom; 3 National Horizons Centre, Darlington, United Kingdom

**Keywords:** leishmania, kinome, protein kinase, activity-based protein profiling, chemoproteomics, drug target, parasite, neglected tropical disease

## Abstract

**Background:**

*Leishmania* parasites cause neglected tropical diseases such as cutaneous and visceral leishmaniasis, which have limited treatment options and rising drug resistance. Protein kinases are pivotal in *Leishmania* biology and attractive drug targets, but their functional status in the parasite remains largely unexplored.

**Methods:**

We applied activity-based protein profiling (ABPP) with custom in-house cell-permeable ATP-site directed probes to map the ligandable, “active kinome” of *Leishmania mexicana*. Three related covalent probes featuring an ATP-mimetic scaffold, electrophilic warhead (targeting catalytic lysines or cysteines), and alkyne tag were synthesised to broadly capture active kinases. Live parasites were labelled with probes, followed by click-chemistry tagging, in-gel fluorescence, and tandem mass tag (TMT) proteomics for kinase identification and quantification. Comparative profiling was performed across *Leishmania* species and life stages. Key findings were validated by competition experiments with ibrutinib and parasite viability assays.

**Results:**

We uncovered 16 metabolic kinases and 32 protein kinases spanning all major kinase families (CMGC, AGC, STE, CAMK, CK1, and NEK), including nine protein kinase enzymes encoded by essential genes and several kinases lacking human orthologs. Notable hits included CRK1, MPK4, CK1.2, and an atypical kinase, underscoring their potential as drug targets.

**Conclusion:**

This study provides the first ABPP survey of the *Leishmania* kinome, revealing multiple ligandable, active kinases that drive parasite survival and virulence. Our chemoproteomic approach highlights both essential protein kinases and unique metabolic kinases as a rich source of potential drug targets. These findings demonstrate that ABPP can unveil the biochemically active kinases in *Leishmania*, offering a new strategy for prioritizing kinase targets and accelerating kinase inhibitor development against leishmaniasis. The work lays a foundation for next-generation antileishmanial therapies directed at the parasite kinome, particularly those kinases indispensable for the parasite yet sufficiently divergent from the human host.

## 1 Introduction

Leishmaniasis, caused by protozoan parasites of the genus *Leishmania*, remains one of the most devastating neglected tropical diseases (NTDs) worldwide. *Leishmania* parasites are transmitted by infected sand flies (*Phlebotomus* and *Lutzomyia* spp.) and alternate between a flagellated promastigote in the insect midgut and an intracellular amastigote that survives within phagolysosomes of mammalian macrophages​. This digenetic life cycle, coupled with diverse parasite species and host immune factors, leads to a spectrum of clinical outcomes. It manifests in cutaneous, mucocutaneous, and visceral forms, with an estimated 0.9–1.6 million new cases and 20,000–50,000 deaths annually (World Health Organization, 2023). Current chemotherapies (including pentavalent antimonials, amphotericin B, miltefosine, and paromomycin) are limited by toxicity, variable efficacy, difficult administration, emerging resistance, and high cost. No effective human vaccine exists, creating an urgent need for new therapeutic strategies targeting crucial parasite pathways (Coutinho de Oliveira et al., 2020)​.

Protein kinases (PKs) have emerged as promising targets in *Leishmania* biology and drug discovery​ (Jones et al., 2018; Efstathiou and Smirlis, 2021). Protein kinases orchestrate phosphorylation-driven signalling and regulate essential cellular processes such as cell cycle progression, differentiation, stress responses, and virulence. The *Leishmania* kinome comprises approximately 175–195 protein kinases, about 2% of the proteome (Efstathiou and Smirlis, 2021). Whilst these kinases span most major groups (CMGC, AGC, CAMK, CK1, STE, NEK, and others), notably *Leishmania* lacks the conventional receptor tyrosine kinases (TKs) and tyrosine-kinase-like (TKL) families found in higher eukaryotes. Instead, *Leishmania* kinomes feature expansions in other families, for example, multiple cyclin-dependent kinases (CRKs) and NEK kinases, reflecting adaptations to its dual host lifestyle (Parsons et al., 2005). Despite their abundance, fewer than 10% of *Leishmania* kinases have been functionally characterised to date (Baker et al., 2021). Nevertheless, several studies underscore their importance (Bhattacharjee et al., 2024; Cayla et al., 2022; Varela-M et al., 2017). For instance, mitogen-activated protein kinase 1 (MPK1) is required for amastigote survival in macrophages (Wiese, 1998), MPK4 and MPK7 regulate differentiation and virulence (Morales et al., 2010), CRK3 (a CDK1 ortholog) is essential for cell cycle progression (Duncan et al., 2016), whilst CRK12 has been validated as a drug target in visceral leishmaniasis (Wyllie et al., 2018). More globally, a kinome-wide CRISPR-Cas9 loss-of-function screens in *Leishmania mexicana* found that ∼44 kinase genes are refractory to deletion suggesting essentiality, reinforcing the fact that many kinases are indispensable for parasite replication, differentiation, or infectivity (Baker et al., 2021). Collectively, these findings indicate that *Leishmania* kinases are critical for parasite survival and infection, highlighting them as attractive candidates for therapeutic intervention. Importantly, parasite kinases often diverge from those of the human host, potentially enabling selective targeting (Merritt et al., 2014). Multiple approaches, including comparative genomics (Parsons et al., 2005; Borba et al., 2019), genetic (Baker et al., 2021), and phenotypic drug screens (Sunkari et al., 2022; Rachidi et al., 2014; Sanderson et al., 2014), have been employed to characterize the *Leishmania* kinome and prioritize kinases as drug targets. Each approach, however, has limitations. For instance, genomic and genetic data identify which kinases are present or essential, but do not reveal which are active under specific conditions; likewise, phenotypic inhibitor screens indicate compounds that affect parasites but do not identify targets. Consequently, identifying which parasite kinases are functionally active (especially in disease-relevant stages) and addressable by inhibitors represents a major knowledge gap for prioritizing kinases in drug development.

Activity-Based Protein Profiling (ABPP) offers a powerful strategy to directly profile enzymatic activity on a proteome-wide scale (Porta and Steel, 2023). ABPP uses small-molecule probes that covalently label enzyme active sites in complex samples, effectively “tagging” enzymes that are catalytically active (Porta, 2023; Porta et al., 2025). Unlike traditional proteomics, which measure protein abundance, ABPP enriches proteins based on activity, reporting which enzymes are active under physiological or near-physiological conditions (Galmozzi et al., 2014)​. As kinases often require specific phosphorylation or allosteric events to become active and expression alone may not reflect functional activity this approach is especially powerful for studying this enzyme class. By using active-site-directed probes that target the conserved ATP-binding pocket, ABPP can capture the subset of kinases in an “active”, ligandable conformation. Studies in mammalian cells have shown that nucleotide analogue probes with electrophilic traps can covalently modify a broad swath of kinases (>75% of the human kinome) by reacting with conserved nucleophilic residues in the ATP site (Patricelli et al., 2007) in cell lysates. Only active kinases, those capable of binding the probe become labelled. The result is a snapshot of the “active kinome” under the tested conditions.

In our previous study (Porta et al., 2023), using highly sensitive TMT-based quantitative proteomics with phosphoproteome enrichment, 1,833 phosphoproteins were identified across the life cycle of *L. mexicana*. The protein kinase domain was the most enriched among phosphorylated proteins, underscoring the pivotal role of phosphorylation in parasite biology and the need for functional approaches to assess kinase activity directly. Herein, we present the first activity-based chemoproteomic profiling of the *Leishmania* kinome. We have synthesised a set of ATP-site covalent probes and applied them to target *Leishmania* protein kinases activity in live parasites. Using comparative in-gel ABPP assays and quantitative mass spectrometry, we mapped the probe responsive *Leishmania* kinome examining how it changes across parasite species and life stages. Our approach uncovered multiple active kinases including many essential and previously uncharacterised enzymes. We compare our findings to earlier genetic and bioinformatic studies and demonstrate how ABPP complements these approaches by highlighting kinases that are biochemically active drug targets. This work introduces ABPP as an innovative tool for kinetoplastid research, providing the first functional atlas of *Leishmania* kinases and a foundation for kinase-targeted drug discovery against leishmaniasis.

## 2 Results

### 2.1 Design of ATP-Site activity-based probes

**FIGURE 1:**
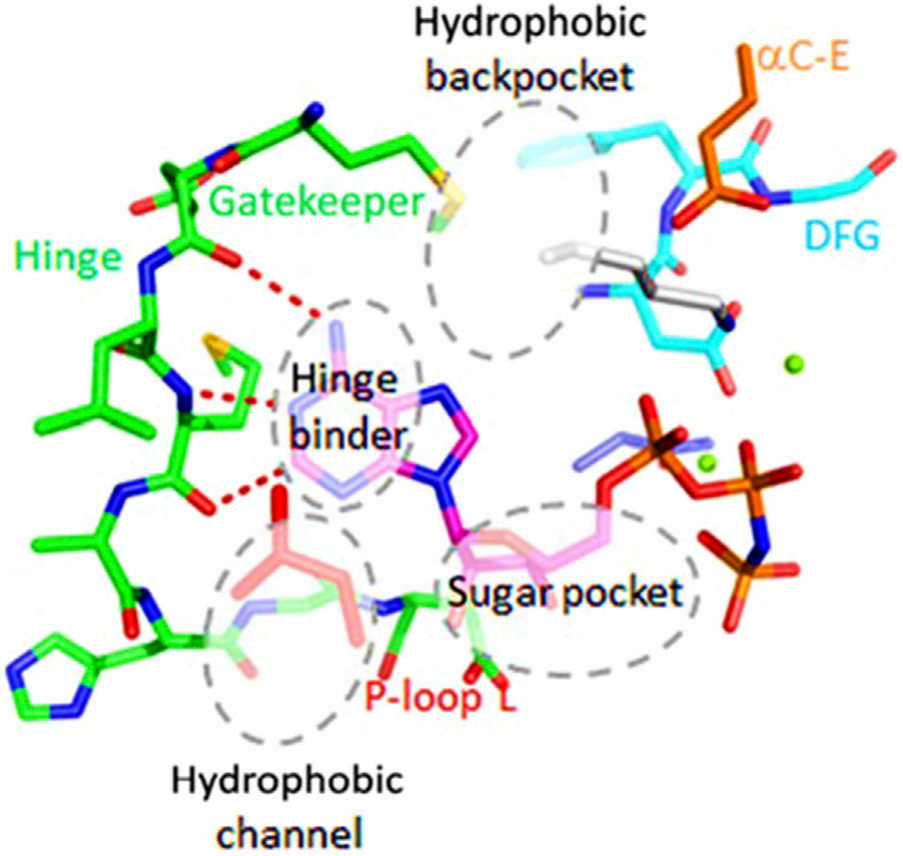
Structure of a prototypical protein kinase domain, highlighting the ATP-binding pocket and surrounding conserved motifs (PDB ID 1GAG).

**FIGURE 2:**
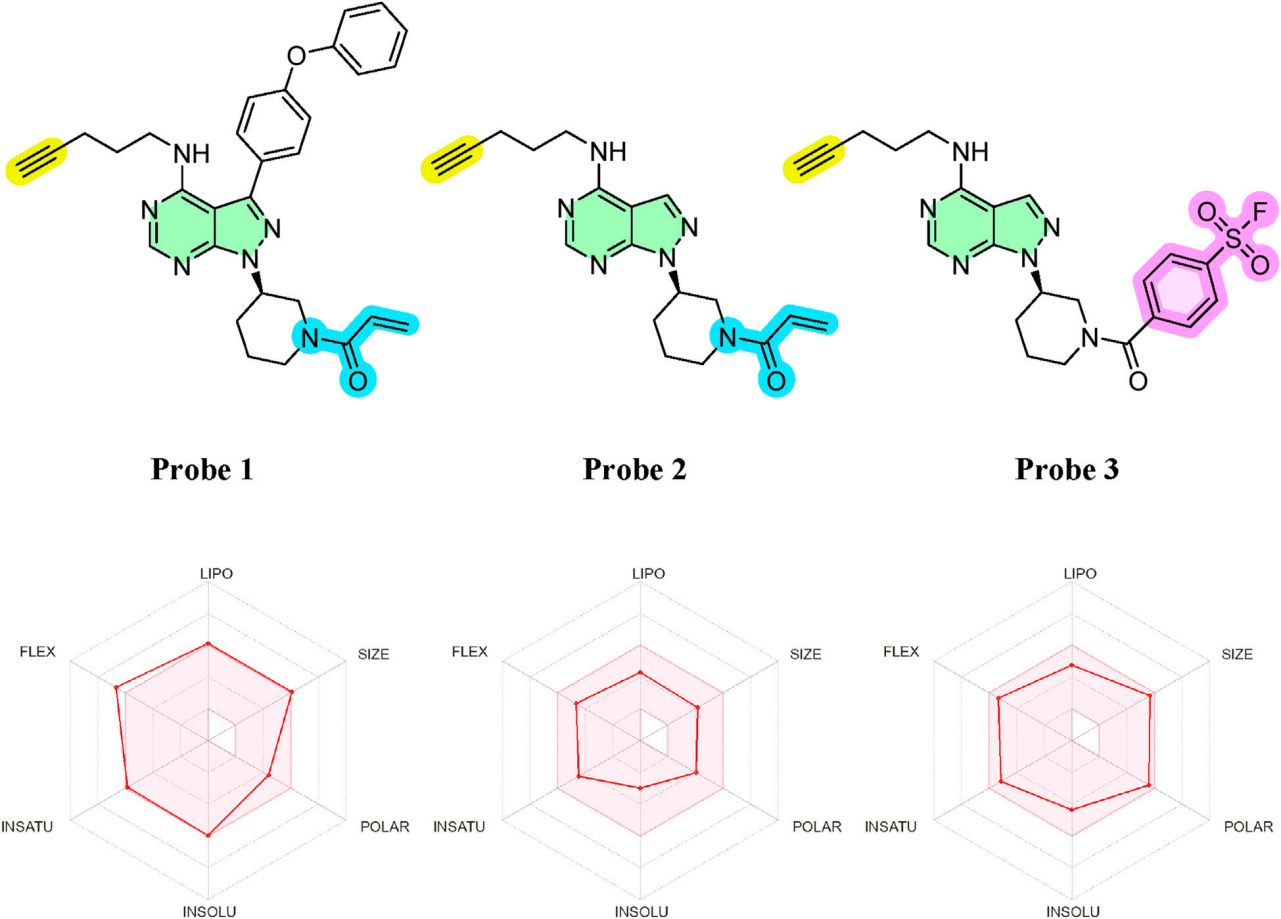
Top: Chemical structures of the three synthesised probes. Highlighted in yellow: biorthogonal alkyne; in green: common nucleobase scaffold; in blue: acrylamide-based warhead; and in pink: arylfluorosulfonate-based warhead. Bottom: *In silico* physicochemical properties of the probes, evaluated using SwissADME (Daina et al., 2017), plotted within the bioavailability radar. The coloured zone represents the optimal physicochemical space for membrane permeability, defined by the following parameters: lipophilicity (−0.7 < Log P < +5.0), molecular weight (150–500 g/mol), polarity (20–130 Å^2^ TPSA), solubility (−6 < Log S < 0), insaturation (Fraction Csp^3^ between 0.25 and 1), and flexibility (number of rotatable bonds <9). The probes were designed to facilitate membrane permeability, with all parameters falling within ranges compatible with intracellular access and biological activity.

The ATP binding pocket of protein kinases is a highly conserved structural feature located at the interface between the N- and C-terminal lobes (Arter et al., 2022) (Figure 1). It includes key elements such as the glycine-rich P-loop, which coordinates the phosphate groups of ATP; the hinge region, which forms hydrogen bonds with the adenine moiety; and the catalytic loop and activation segment, containing critical residues like the conserved catalytic lysine and the DFG motif that regulate activity. This pocket not only supports the hydrophobic interactions required to secure the adenine ring of ATP but also displays subtle differences across kinases that allow for selective inhibitor binding. These conserved yet distinct structural features make the ATP binding pocket an ideal target for designing activity-based probes (ABPs) aimed at covalently labelling active kinases, thus enabling a functional readout of kinase activity in *Leishmania*. Based on this, we developed three ABPs to broadly target *Leishmania* protein kinases (Figure 2). Each probe consisted of three modular components: (1) an electrophilic warhead designed to covalently react with nucleophilic amino acid residues in kinase active sites, (2) an ATP-mimetic scaffold to direct the probe to the conserved ATP-binding pocket, and (3) a biorthogonal reporter handle (terminal alkyne) for downstream conjugation and detection​. The overall design was inspired by known covalent kinase inhibitors. In particular, we leveraged the scaffold of ibrutinib (Davids and Brown, 2014), a potent human Bruton’s tyrosine kinase (BTK) inhibitor, as a starting point, which contains an acrylamide warhead designed to bind to the Cys481 residue in the active site of the BTK protein. Whilst ibrutinib binds tightly in the ATP-binding pocket of BTK (Roskoski, 2016) and, in a clinical setting, is exquisitely selective for BTK, at higher concentrations it begins to lose selectivity, affecting other kinases and other proteins (Nicolson et al., 2018) and as such resembles an ABP.

### 2.2 Probe synthesis

**SCHEME 1:**
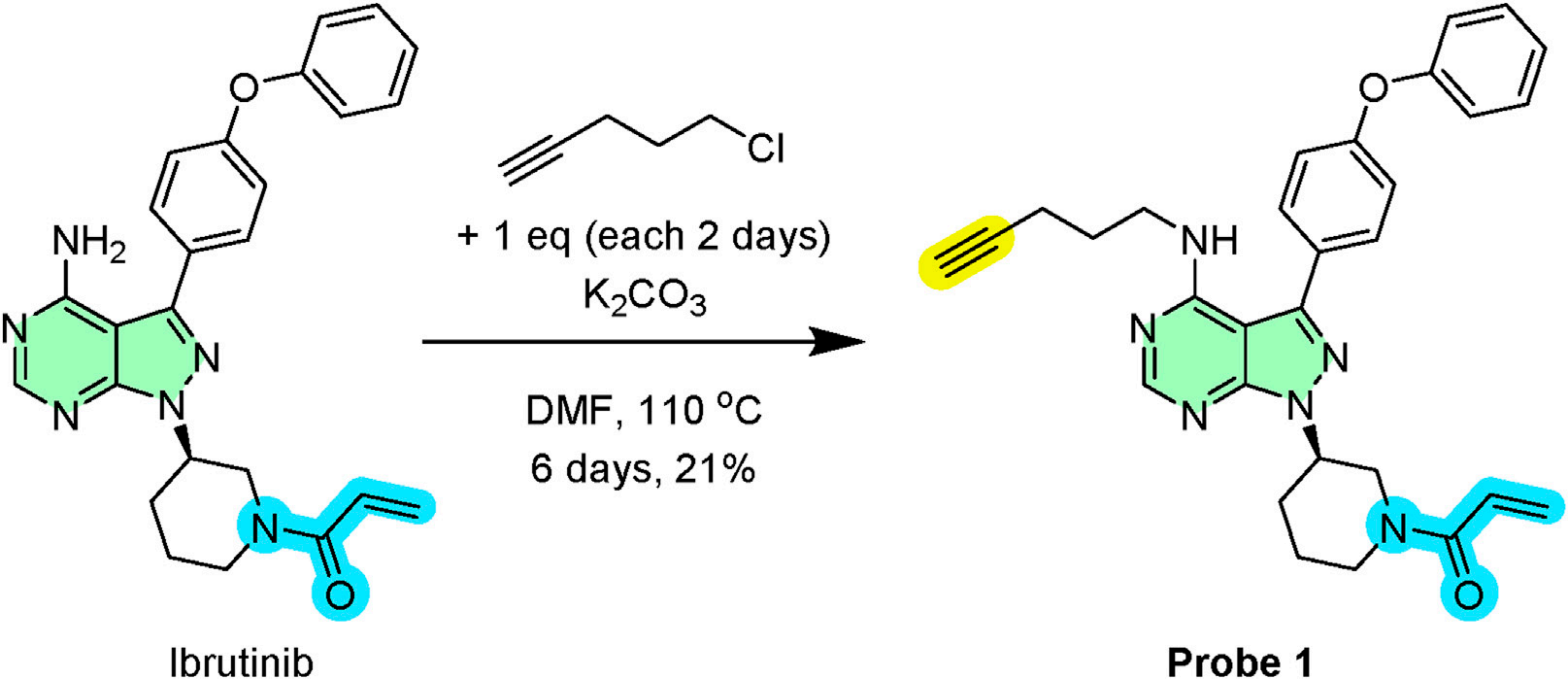
Synthetic route for probe 1.

Initially, we directly functionalised ibrutinib’s core to append the biorthogonal alkyne handle (Scheme 1). In this, ibrutinib was N-alkylated with 5-chloropentyne under mildly basic conditions to afford the desired alkyne-conjugated probe (1) in 21% yield after purification. The stability of Probe 1 was verified by preparing a 1 mM solution and analysing it via NMR and LC-MS; no degradation was detected in either PBS or DMSO after 20 h at 37 °C. Furthermore, when stored as a 10 mM solution in DMSO at −20 °C, the probe remained intact for over 3 months (Supplementary Figure S1).

**SCHEME 2:**
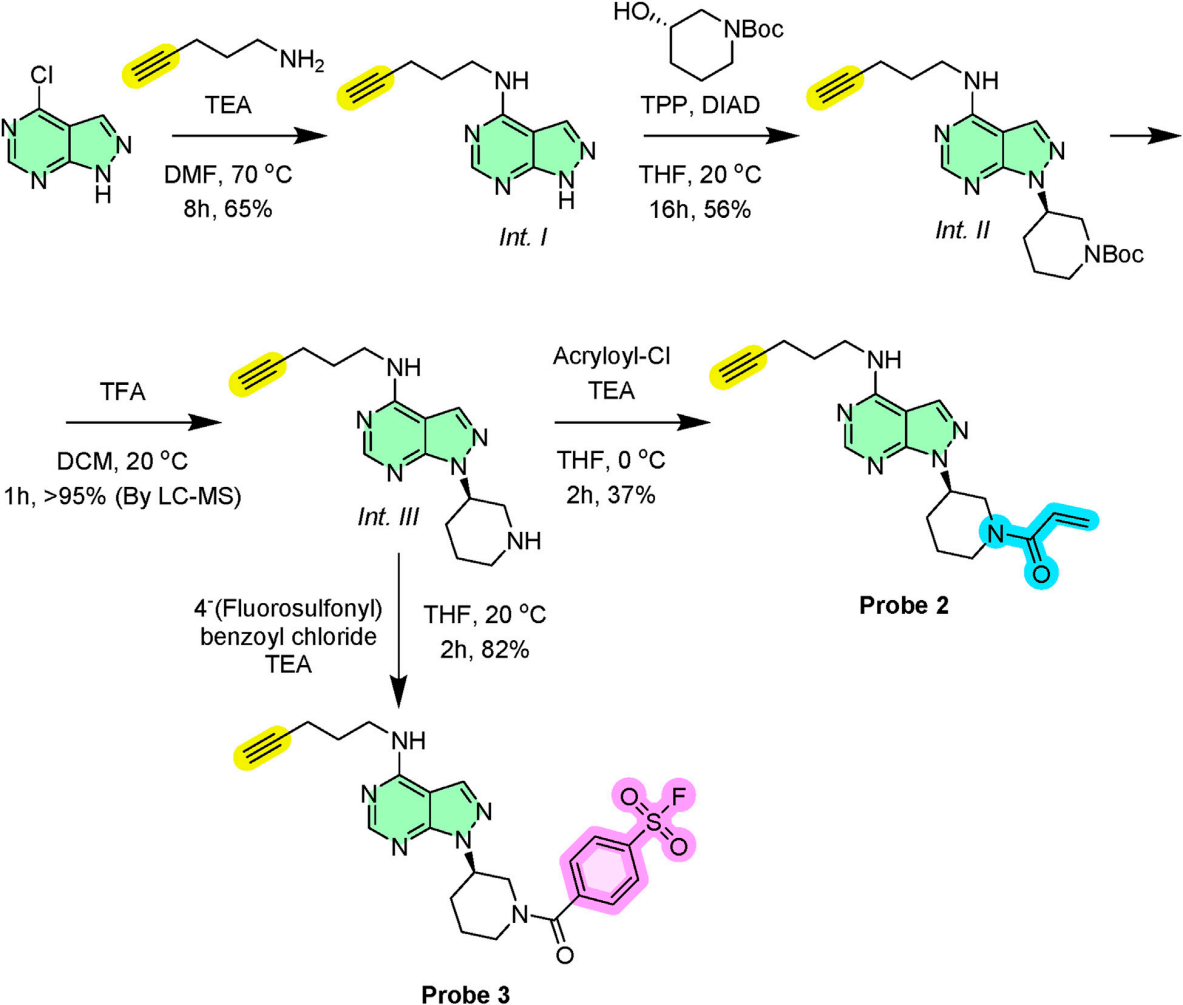
Synthetic route for probe 2 and probe 3.

To increase pan-kinase coverage, we then synthesised two variants of a simplified scaffold lacking the bis-aryl ether motif that defines ibrutinib’s BTK selectivity. Starting from commercially available chloro-substituted pyrimidine core, we introduced the alkyne handle via nucleophilic aromatic substitution (S_N_Ar) with propargylamine (*Int. I*) (Scheme 2). The key protected secondary amine (*Int. II*) linkage forming a hinge-binding motif and a sugar pocket analogous to ibrutinib was introduced via Mitsunobu chemistry. Finally following carbamate deprotection (*Int. III*), acylation with acryloyl chloride gave Probe 2 targeting cysteine nucleophiles (Wang et al., 2025). Alternatively, acylation with *p*-fluorosulfonyl benzoylchloride afforded Probe 3 in which the sulfonyl fluoride favours reaction with lysine or serine residues​ (Gilbert et al., 2023).

### 2.3 Broad-spectrum probes label diverse kinases in *Leishmania mexicana*

**FIGURE 3:**
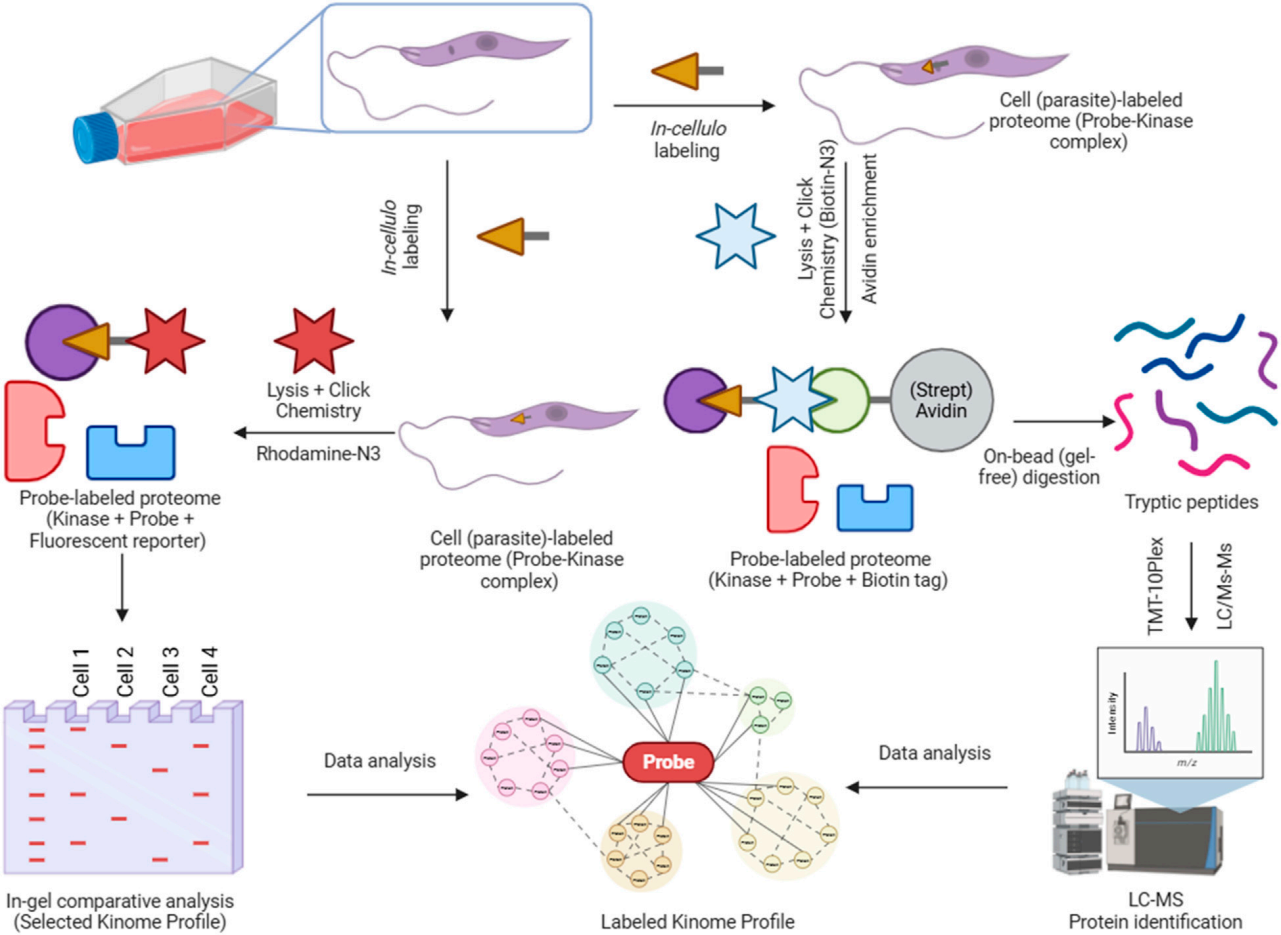
An activity-based protein profiling (ABPP) workflow for target (kinome) identification in *Leishmania mexicana*. Live *L. mexicana* parasites are incubated with an alkyne-functionalised activity-based probe (e.g., Probe 1, 2, or 3) to covalently label target proteins. Following lysis, probe-labelled proteins are conjugated to an azide reporter tag via CuAAC (“click chemistry”) for two distinct downstream applications. For visualisation, a fluorescent tag (Rhodamine-N_3_) is used for analysis by SDS-PAGE and in-gel fluorescence scanning. For proteomic identification, a biotin-N_3_ tag is used to enrich target proteins (kinases) via affinity purification. These enriched proteins are then digested, labelled with Tandem Mass Tags (TMT) for multiplexed quantification, and analysed by LC-MS/MS.

**FIGURE 4:**
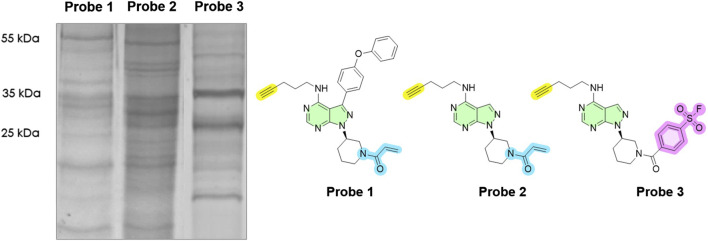
In-gel fluorescence analysis (with emission detected at 580 nm) of *Leishmania mexicana* at a final protein concentration of 1 mg/mL revealed an active protein fingerprint upon treatment with our 10 μM probes. Lane 1: Probe 1; Lane 2: Probe 2; Lane 3: Probe 3. Typhoon laser scanner (excitation 532 nm, emission 580 nm) and ImageQuant TL software (normal sensitivity and PMT 500 or 600 V; GE Healthcare Life Sciences). DMSO (no-probe) controls are shown in Supplementary Figure S3.

To profile the active kinome of *Leishmania* (ABPP workflow described in Figure 3), we first examined labelling patterns of Probe 1–3 in *L. mexicana* promastigotes (*in cellulo*). Based on the dose–response (0–20 µM), labelling increased and then saturated near 10–20 μM; we therefore selected 10 µM as a tracer-like concentration that delivers strong coverage without additional gains at higher doses (Supplementary Figure S2). Therefore, parasite cells were treated with each probe (10 μM at 26 °C for 2 h) and lysed. Samples were click-conjugated to the fluorescent Tetramethylrhodamine (TAMRA)-azide reporter and analysed by SDS-PAGE. All three probes yielded prominent fluorescent bands, indicating successful covalent labelling of parasite proteins. Because single fluorescent bands can contain multiple proteins (Porta and Steel, 2023), we interpret the gels as aggregate activity profiles by molecular-weight region. Thus, the in-gel assay is used primarily for qualitative comparison; i.e., distinct fingerprints indicate different patterns of probe engagement/activities across experimental conditions. The overall ABPP “fingerprint” consisted of multiple bands with different molecular weights (Figure 4). Notably, the banding patterns were highly enriched for sizes corresponding to proteins mostly in the range 20–60 kDa. Strong bands were observed around 25–35 kDa, and 50–60 kDa, consistent with the molecular weights of typical kinases. Each probe showed a different labelling profile, reflecting their differing structural specificities. The acrylamide-based probes (probes 1 and 2) gave broadly similar patterns (Supplementary Figure S3), whereas the sulfonyl fluoride probe (probe 3) showed relatively enhanced labelling of certain bands (e.g., intense bands at ∼35 kDa and ∼25 kDa) and slightly reduced labelling of others (e.g., at ∼20 kDa). When comparing probes directly, any one probe alone captured a large portion of the bands observed with the others. This suggests that each probe independently labels a broad set of molecular targets and combining them ensures a broader coverage of kinase active sites in *Leishmania*.

### 2.4 Competitive ABPP

**FIGURE 5:**
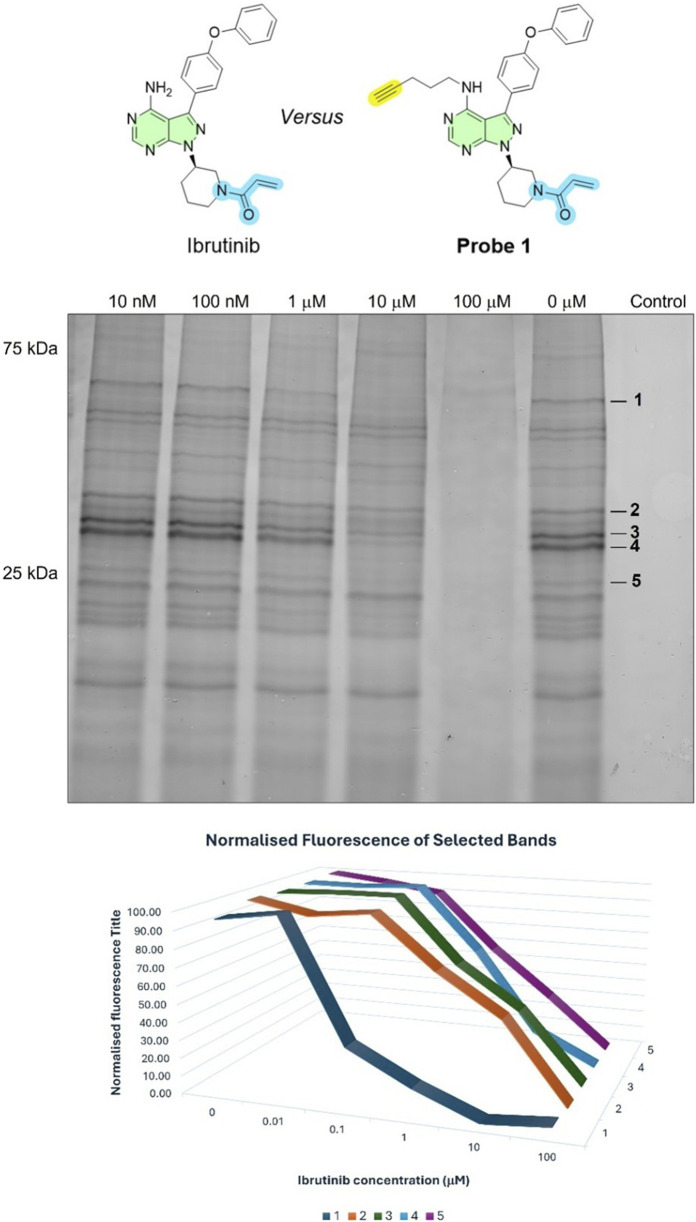
Competitive ABPP with ibrutinib in *Leishmania mexicana* promastigotes. Top: In-gel fluorescence after live-cell labelling with Probe 1 (10 μM, 2 h) in the presence of ibrutinib (0–100 μM; pre-incubated for 4 h and present during labelling). DMSO is the vehicle (0 µM) and the no-probe control and the final protein concentration is 1 mg/mL. Right margin marks selected bands one to five quantified below. Bottom: Normalised fluorescence for bands 1–5 as a function of ibrutinib concentration. Dose-dependent competition indicates overlap with the ATP site/ligandable pocket for a subset of targets; bands with little change are ibrutinib-insensitive under these conditions.

To confirm target engagement, live *L. mexicana* promastigotes were pre-incubated with varying concentrations of ibrutinib (EC_50_ = 26 ± 4 μM), our starting point molecule, for 4 h (0, 0.01, 0.1, 1, 10, 100 μM; DMSO-control), followed by labelling with 10 μM Probe 1 for 2 h at 26 °C. Consistent with competitive binding a reduction in the fluorescence intensity of multiple probe-labelled bands on the in-gel fluorescence readout (notably bands 25–35 kDa and 55–65 kDa) compared to a no-inhibitor (DMSO) sample could be observed (Figure 5). Normalised fluorescence of selected bands showed monotonic competition when normalised to the probe-only condition, with near-complete loss at high ibrutinib (≥10–100 µM). Importantly, no novel bands appeared, and the global labelling pattern remained comparable to control (0 μM, DMSO), indicating target-specific engagement rather than pathway-level rewiring during the ibrutinib 4-h exposure. At concentration of 100 μM, ibrutinib caused lysis in the parasites. These results confirm that ibrutinib competes in a dose dependent fashion with Probe 1 for binding to active kinase sites in *L. mexicana*, validating the specificity of our labelling approach. Similarly, the findings suggest that ibrutinib mediates its antileishmanial activity by inhibiting multiple targets.

### 2.5 Cross-species comparison of profiles

**FIGURE 6:**
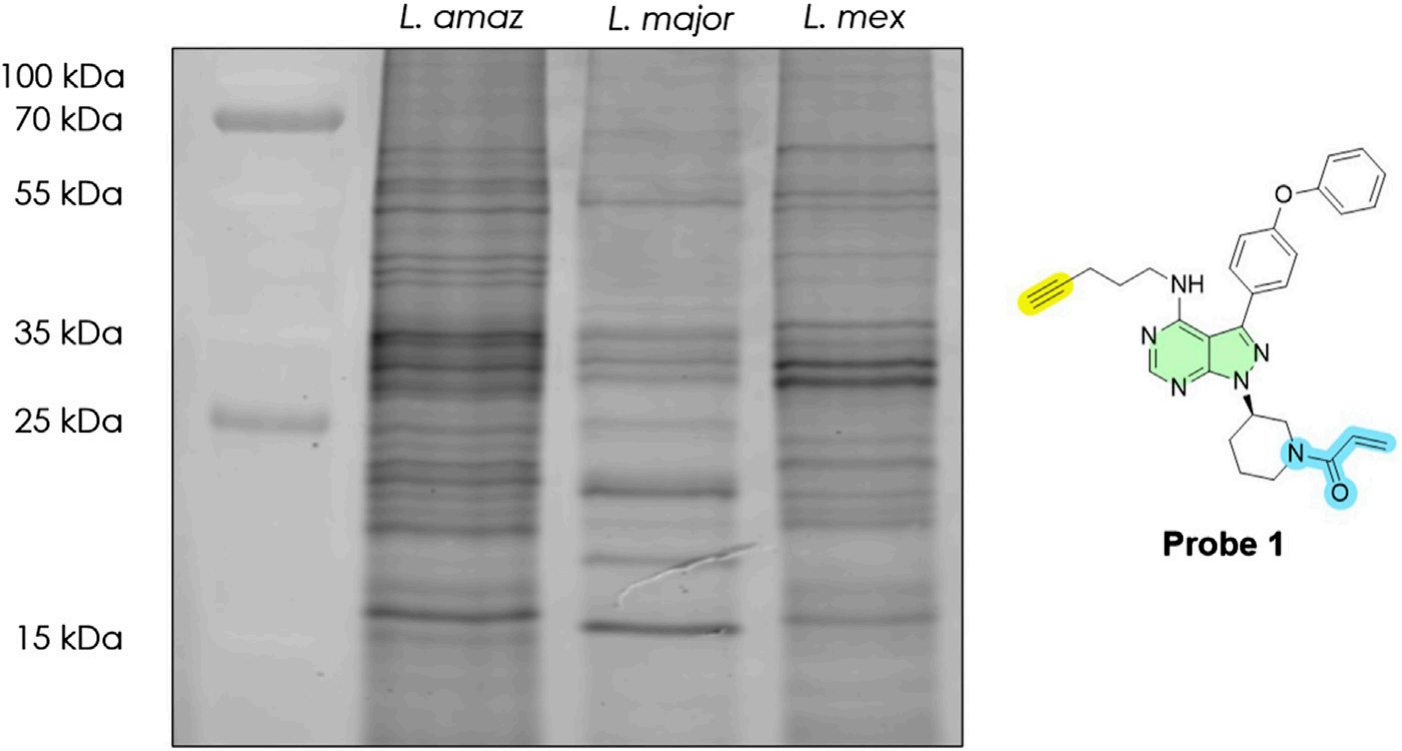
In-gel fluorescence analysis (emission at 580 nm), active molecular target fingerprint of selected *Leishmania* spp. (final protein concentration: 1 mg/mL) revealed by Probe 1 at 10 μM. Lane 1: Ladder; Lane 2: *L. amazonensis*; Lane 3: *L. major*; Lane 4: *Leishmania mexicana*. Typhoon laser scanner (excitation 532 nm, emission 580 nm) and ImageQuant TL software (normal sensitivity and PMT 500 or 600 V; GE Healthcare Life Sciences). DMSO (no-probe) controls are shown in Supplementary Figure S4.

While the kinase gene complement is largely conserved across *Leishmania* species, we then asked whether different species exhibit distinct active (kinome) profiles. To explore this, we applied our ABPP in-gel assay to compare three species: *L. mexicana*, *Leishmania amazonensis*, and *Leishmania major*, representing both ‘New-World’ and ‘Old-World’ strains. Equal amounts of procyclic promastigote cells from each species were labelled with Probe 1 under identical conditions (10 μM for 2 h), lysed, decorated with TAMRA-azide, and analysed side-by-side on an in-gel fluorescence gel (Figure 6). All three species showed multiple fluorescent bands across a wide molecular weight range (15–100 kDa), indicating active proteins in each. However, the banding patterns were not identical, revealing species-specific differences in active molecular targets.

Certain bands appeared common to all species, for example, a cluster of bands around 30–35 kDa and a band near 50 kDa were prominent in all three species. In contrast, some signals differed markedly. *L. major* (Old-World strain) displayed two strong bands at ∼20 and ∼15 kDa that were barely detectable in the two New-World species. Conversely, *L. mexicana* showed much stronger labelling of ∼30 kDa bands compared to *L. major*​ (∼3.7-fold greater). These inter-species differences may suggest that while the *Leishmania* kinomes share similar components, the basal activity of certain kinases varies by species. Such variation might correlate with differences in biology or pathology between species. It is also observed that the two New-World species (*L. mexicana* and *L. amazonensis*) had broadly similar profiles relative to the Old-World *L. major*, supporting the notion that closely related species have more similar kinome activity patterns (consistent with their phylogenetic grouping). Similar observations could also be seen in related studies exploring serine hydrolases activity (Porta et al., 2022; Isern et al., 2025). These findings, albeit based on gel profiles, demonstrate that ABPP can discern species-specific protein activity “fingerprints”. They hint that even subtle differences in protein regulation among *Leishmania* can be captured by functional profiling, an aspect that purely genomic comparisons (which show, for example, ∼90% kinase gene conservation across species) would miss.

### 2.6 Stage-specific profiles in the *Leishmania* life cycle

**FIGURE 7:**
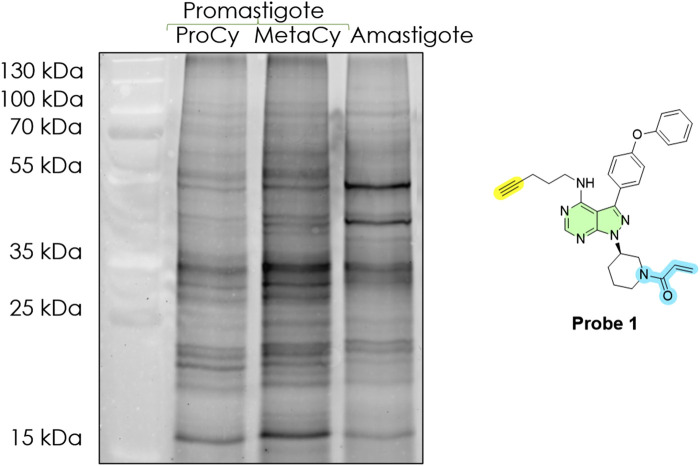
In-gel fluorescence analysis (emission at 580 nm), active molecular target fingerprint of three life cycle stages of *L. amazonensis* (final protein concentration: 1 mg/mL), revealed by Probe 1 at 10 μM. Lane 1: Ladder; Lane 2: procyclic promastigotes (ProCy); Lane 3: metacyclic promastigotes (MetaCy); Lane 4: axenic amastigotes (amastigote). Typhoon laser scanner (excitation 532 nm, emission 580 nm) and ImageQuant TL software (normal sensitivity and PMT 500 or 600 V; GE Healthcare Life Sciences). DMSO (no-probe) controls are shown in Supplementary Figure S4.

We next examined how the active profile of *Leishmania* changes during the parasite’s life cycle. Using *L. amazonensis* as a model procyclic promastigote, metacyclic promastigote, and axenic amastigote, cells were analysed using probe 1, lysed, tagged with azido TAMRA, and subsequently resolved by SDS-PAGE (Figure 7). The results revealed marked stage-specific differences in protein activity.

Procyclic promastigotes, the midgut form in the sand fly, analogous to logarithmic-phase culture promastigotes, showed a relatively simple gel with few dominant fluorescent bands, primarily in the 20–25 kDa and 25–35 kDa ranges and bands at 50–55 kDa​. This suggests that in rapidly dividing promastigotes, only a subset of proteins is highly active. Metacyclic promastigotes, the actual infectious agent, displayed an increase in both intensity and number of bands, especially in the 25–35 kDa region. Several bands that were faint or absent in procyclics became strongly labelled in metacyclics​. Notably, a band around ∼35 kDa was much more pronounced in metacyclics than procyclics (∼4.6-fold greater)​. The overall fluorescence signal was higher in metacyclics, indicating an upregulation in enzyme activities.

The amastigote lane showed a distinct profile different from both promastigote forms​. We observed two prominent bands at ∼50 kDa and ∼45 kDa that were either very faint or not present in promastigotes​. Conversely, bands prominent in promastigotes (∼35 kDa and ∼15 kDa) were reduced in amastigotes to ∼28% and ∼21% of the promastigote signal, respectively. Others (such as the ∼55 kDa band) were nearly undetectable or absent. This indicates those proteins are downregulated or inactive in the intracellular stage and is consistent with earlier reports that lower numbers of proteins are expressed in the amastigote stage (Morales et al., 2008).

### 2.7 Chemoproteomic identification of active kinome components

Having established robust labelling, next, we identified the probe-tagged proteins employing quantitative mass spectrometry. Using *L. mexicana* as the model species with well-established genomic data coupled with information available regarding the essentiality of the protein kinase (Baker et al., 2021). Promastigotes were labelled with each probe (with parallel DMSO-treated controls), and probe-bound proteins were affinity-enriched and analysed by LC-MS/MS with Tandem Mass Tag (TMT) labelling quantification.

**FIGURE 8:**
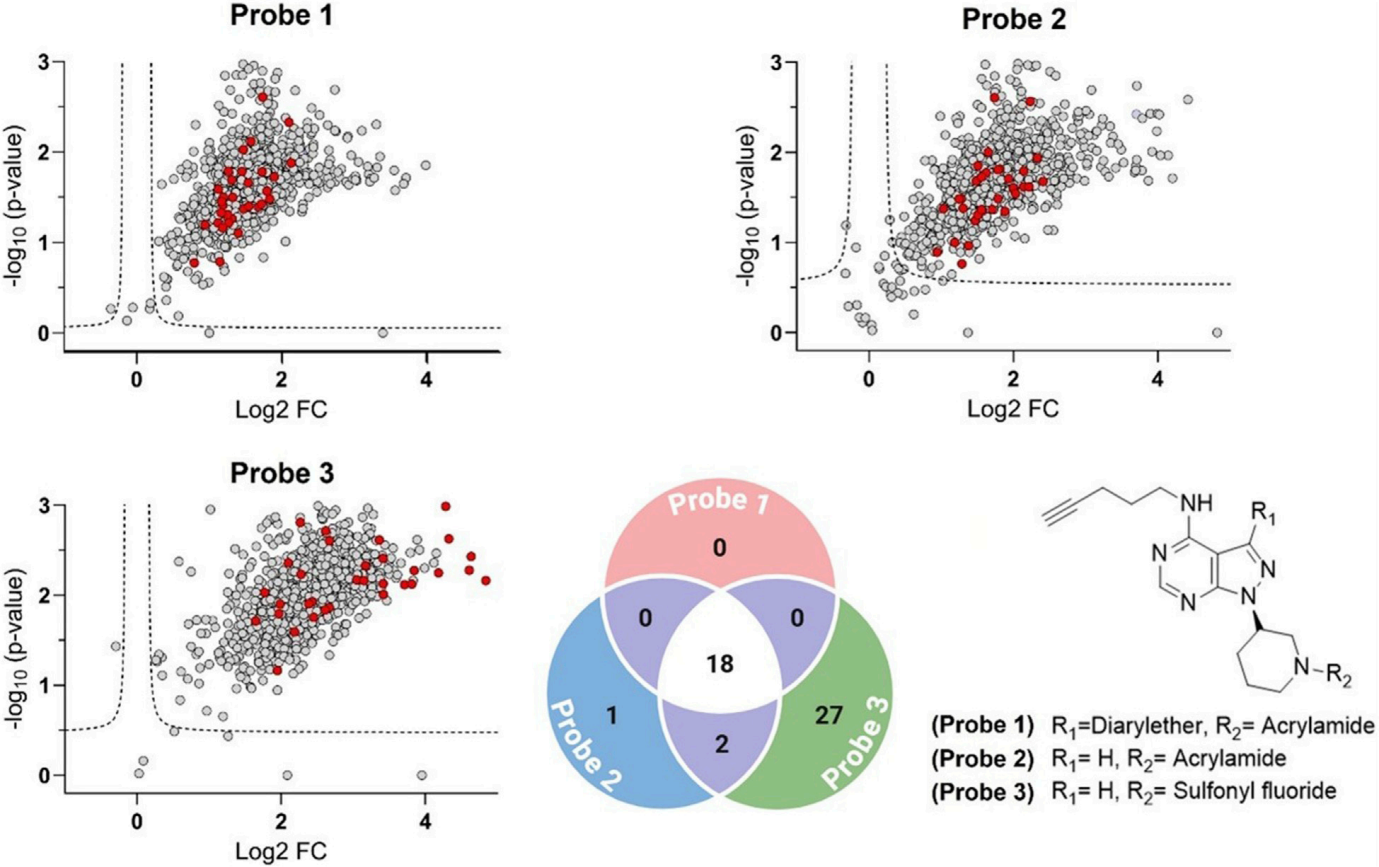
Volcano plots of ABPs. Labelling performance of the three activity-based probes (ABPs) at 10 µM in *Leishmania mexicana* promastigotes (final protein concentration of 1.5 mg/mL) was quantitatively compared with no-probe treatments (DMSO) using activity-based protein profiling (ABPP) followed by tandem-mass-tagging (TMT)-labelling-based quantitative proteomic mass spectrometry (MS). All experiments were performed in biological replicates. A modified *t*-test with permutation-based FDR statistics (250 permutations, FDR = 0.05) was applied to compare probe treatments versus control groups. In the volcano plots, the x-axis represents enrichment on a log_2_ scale, while the y-axis shows the -log_10_ p value. Proteins were considered significantly enriched if they satisfied q < 0.05 (permutation-based FDR <5%) and log_2_ FC ≥ 0.5. The dashed lines indicate these thresholds. The asymmetric shape of the plots reflects enrichment over DMSO controls, as expected for activity-based probes. Positive protein kinases of the probes that passed these cut-off values are shown on the right-hand side as red-filled circles, with the identified *Leishmania mexicana* protein kinome annotated using their UniProt accession numbers. The curated kinases are listed in Supplementary Table S1. Probe 3 showed superior enrichment of the full set of detected protein kinases compared to Probe 1 and 2 (as shown in the volcano plot). Venn diagram (bottom) summarising kinase counts (protein kinases plus ATP/GTP-dependent metabolic kinases) that are significantly enriched for each probe (q < 0.05, permutation-based FDR) and meet a stricter Δlog_2_FC ≥ 1 (≥2-fold) effect-size threshold. Δlog_2_FC (Probe X, Probe Y) = log_2_FC (Probe X vs. DMSO) – log_2_FC (Probe Y vs. DMSO) (e.g., Probe 3 − Probe 2). Numbers in the non-overlapping sectors denote kinases uniquely captured by a single probe; overlapping sectors denote kinases shared between probes.

**FIGURE 9:**
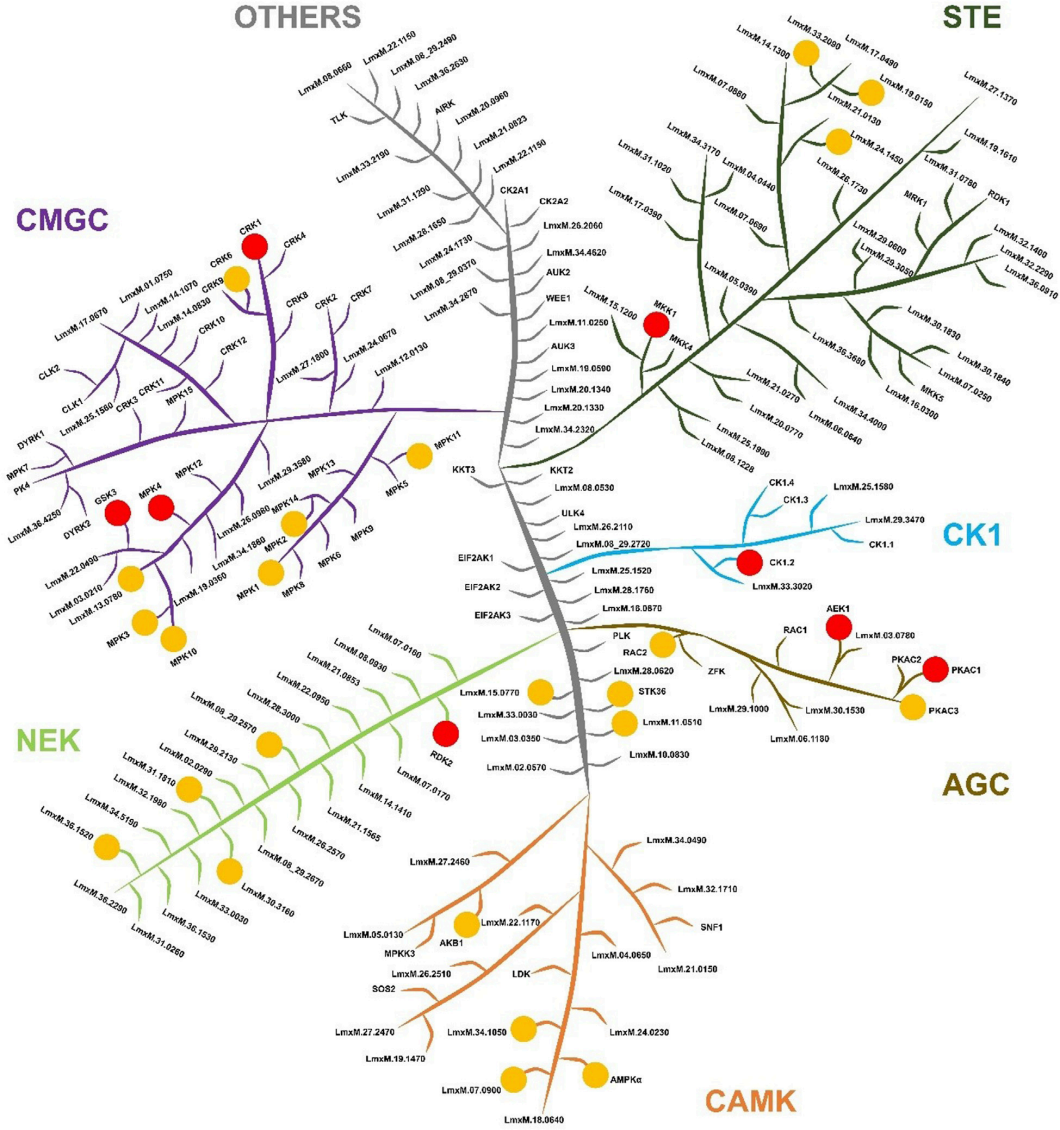
Labelled protein kinases in *Leishmania mexicana* eukaryotic protein kinome. Classification of the protein kinases in *Leishmania mexicana* according to their catalytic domain types, namely, CMGC, STE, NEK, CK1, AGC, CAMK, and others. The image has no phylogenetic significance and is for illustrative purpose only. Red dots represent the labelled PK for which gene deletion mutants could not be generated and are therefore potentially essential genes in procyclic promastigotes (Baker et al., 2021). The yellow dots represent labelled protein kinases in which gene deletion mutants could be generated; therefore, potentially non-essential genes.

Proteins were considered significantly enriched if they satisfied q < 0.05 (permutation-based False Discovery Rate <5%) and log_2_ fold change (log_2_ FC) ≥ 0.5. Combining results from all three probes, we identified a total of 657 parasite proteins significantly enriched by probe treatment relative to the DMSO control. The asymmetric shape of the plots reflects enrichment over DMSO controls, as expected for ABPs (Figure 8). These represent the putative “targets” of our ATP-site probes. As expected, kinases were the largest functional category among enriched targets and define the operational active kinome subset used for downstream analyses (Supplementary Table S1). More globally these broad-spectrum probes demonstrate good selectivity for nucleotide binding or interaction sites. For example, among the top five protein families, each has either nucleotide binding sites or sites for interacting with RNA or DNA (protein kinases, helicases, RNA recognition motif proteins, etc.). Notably, protein kinases were the most strongly captured functional class and showed the largest effect sizes across metrics (including log_2_ FC). Among kinases meeting q < 0.05 (permutation-based FDR), the mean enrichment (probe vs. no-probe/DMSO) was log_2_ FC ∼1.7 for Probe 1 (∼3.2-fold), ∼1.9 for Probe 2 (∼3.7-fold), and ∼3.0 for Probe 3 (∼8-fold). Focusing on the high-confidence hits, we identified 32 proteins that are *bona fide* protein kinases. The remaining kinase-like hits (16 proteins) consisted of other ATP/GTP-dependent metabolic kinases. This suggests that kinases, despite being only ∼2% of the proteome, constitute a dominant portion of the probe targets, likely because they harbour highly reactive ATP sites that avidly bind the probes. To compare probes pairwise, we defined Δlog_2_FC as: Δlog_2_FC(Probe X, Probe Y) = log_2_FC(Probe X vs. DMSO) – log_2_FC(Probe Y vs. DMSO), so positive values indicate stronger enrichment by Probe X, and *vice versa*. Interestingly, Probe 3 showed better enrichment of the complete set of detected protein kinases, compared to Probe 1 and 2. For instance, taking into account all targets, the average Δlog_2_FC values support this ranking: the average Δlog_2_FC for Probe 3 vs. Probe 1 was ∼0.80 and for Probe 3 vs. Probe 2 was ∼0.62, while the average Δlog_2_FC for Probe 2 vs. Probe 1 was only ∼0.18. These data indicate a clear enrichment efficiency ranking of Probe 3 > Probe 2 > Probe 1 (Figure 9). This observation aligns with the well-established conservation of lysine residues within this nucleoside triphosphate (NTP) binding domain (Patricelli et al., 2007; Serafim et al., 2022): probe 3’s broader enrichment is consistent with lysine-selective sulfonyl fluoride chemistry targeting the conserved ATP-site lysine (Gilbert et al., 2023), whereas acrylamide probes favour less common cysteine residues at or near the pocket (Backus et al., 2016; Zhao et al., 2017). The small but consistent Probe 2 > Probe 1 difference (Δlog_2_FC ∼0.18) suggests that removing the diaryl-ether headgroup modestly broadens target compatibility. The 32 active protein kinases span all major kinase families in the parasite kinome (Figure 9). The diversity of kinases captured demonstrates that our ABPP approach using broad-spectrum probes is unbiased with respect to kinase class, enriching representatives from every major clade of the *Leishmania* kinome.

### 2.8 Functional validation of ABP labelling specificity using pyruvate kinase (PYK)

Finally, having established that our ATP-directed ABPs robustly label a diverse spectrum of active kinases in *L. mexicana*, we aimed to validate their specificity towards properly folded, active enzymes. For this, on the basis of strong and consistent enrichment of our chemoproteomic analyses coupled with ready availability of a suitable model, we selected pyruvate kinase (PYK), a metabolic kinase of significant interest due to its essential role in parasite energy metabolism, allosteric regulation, and potential as a selective drug target as a model enzyme for detailed functional validation. Although traditionally recognised as a metabolic enzyme catalysing the final step in glycolysis, recent studies highlight that certain isoforms of PYK, notably PKM2, exhibit moonlighting kinase activity, phosphorylating various protein substrates using either phosphoenolpyruvate (PEP) or ATP as phosphate donors (Lu and Hunter, 2018). Structural analyses support this dual functionality, revealing a well-defined ATP-binding domain containing conserved catalytic motifs (Schormann et al., 2019), further validating the choice of PYK for initial probe validation. Whilst *Leishmania* PYK has been previously described (Ernest et al., 1994; Morgan et al., 2010), for simplicity in this experiment we opted to use commercially available rabbit muscle PYK, which has a high degree of homology with the parasite enzyme (49% identity and 64% similarity to *L. mexicana*), making it a suitable surrogate model for ATP-binding kinase validation (Supplementary Figure S5).

**FIGURE 10:**
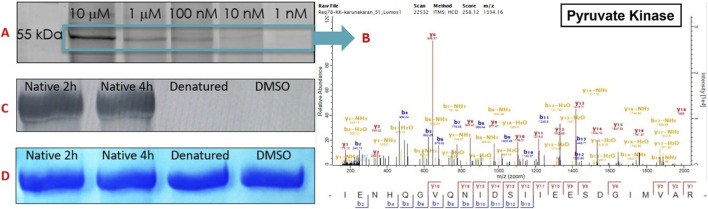
**(A)** Labelling PYK with Probe 1 at variable concentrations. **(B)** MS/MS spectrum of the selected band (55 kDa). **(C)** Labelling PYK with probe 1 (10 μM) in four different conditions (native 2h, native 4h, denatured, and no-probe/DMSO). **(D)** Coomassie blue of **(C)**.

Briefly, rabbit muscle PYK was incubated with probe 1 (10 µM) under three conditions: (1) native PYK, 2 h incubation; (2) native PYK, 4 h incubation; and (3) heat-denatured PYK (95 °C, 5 min), 4 h incubation. A no-probe (DMSO) control was also included. A prominent fluorescent band at ∼55 kDa (PYK) was observed exclusively in the active PYK samples, with similar intensity after 2 h and 4 h of incubation, while the denatured PYK showed no detectable fluorescence. Coomassie staining confirmed equal protein loading across conditions. Furthermore, mass spectrometry analysis (LC-MS/MS) of the excised fluorescent ∼55 kDa band confirmed probe labelling on PYK. These results demonstrate that our probe covalently modifies the enzyme’s ATP-binding pocket only when it is properly folded and active, with negligible nonspecific interactions with denatured protein (Figure 10). Thus, this validation confirms that parasite labelling predominantly reflects genuine active-site engagement: the “active kinome”.

## 3 Discussion

In this study, we present the first activity-based chemoproteomic profiling of the *Leishmania* kinome, providing a functional insight into ligandable, active kinases within the parasite. By using ATP-directed probes, we moved beyond static genome annotation to profile kinases active under physiological conditions. This approach addresses a critical gap in *Leishmania* research, where kinase information traditionally stemmed from sequence homology, biochemical assays, or genetic screens, which do not directly report on catalytic activity. ABPP, however, interrogates kinase function directly, offering a functional representation of the active kinome across life stages and species, rather than mere presence (Porta and Steel, 2023). Therefore, kinome ABPP provides a unique and powerful snapshot of kinase functional status by operationally defining the “active kinome” as the pool of kinases that are ATP-site–accessible and ligandable. By using probes to target the accessibility and conformation of the native active site (often labelling a conserved lysine or cysteine) this technique precisely identifies the fraction of kinases competent to bind ATP-like ligands. This readout directly mirrors cellular target engagement and offers a direct measure of drug targetability. Crucially, ABPP does not measure catalytic rate (turnover) or a specific phospho-state. Instead, it reports on the functionally relevant, ligandable state, a principle that has been widely validated *in situ* to define kinase inhibitor engagement and selectivity windows (Patricelli et al., 2007; Lanning et al., 2014). Therefore, while ABPP assesses this functionally competent state, actual turnover must be confirmed using orthogonal assays.

The effectiveness of our ABPP strategy is highlighted by the identification of 32 protein kinases and 16 metabolic kinases actively labelled in promastigotes, many experimentally uncharacterised. These proteins represent the operational components of the parasite kinase signalling network under the standard culture conditions used and the probes employed. Each kinase identified has a role in parasite biology, from regulating development (e.g., CRK and MAPK families) to maintaining essential metabolic functions (e.g., phosphoglycerate kinase). The enrichment of kinases known or predicted to be essential in *L. mexicana* supports the biological relevance of our ABPP data. For example, CRK1, essential for mitosis, (Cayla et al., 2014; Duncan et al., 2016), is indispensable in promastigotes without a heterologous substitute (Jones et al., 2014). Similarly, MPK4 is crucial for promastigote-to-amastigote differentiation and metacyclogenesis (von Freyend et al., 2010; Baker et al., 2021) with its loss leading to defects in stage transition and reduced virulence (Wiese, 2007). MPK10 represents another kinase identified that is linked to stage adaptation being required for normal promastigote-to-amastigote development (Horjales et al., 2012; Cayla et al., 2014). Finally, CK1.2 has been implicated in parasite viability and virulence, reinforcing its potential as a therapeutic target, supported by the identification of several inhibitors targeting this enzyme (Allocco et al., 2006; Rachidi et al., 2014; Dan-Goor et al., 2013).

This chemoproteomic approach also illuminated the “dark kinome” in *Leishmania* with several active kinases identified having been annotated as hypothetical or uncharacterised proteins. For example, we captured an ABC1 family kinase, suggesting an active metabolic role possibly related to mitochondrial regulation (Lundquist et al., 2012). This highlights the potential of ABPP to uncover functionally significant yet overlooked proteins (kinases) that might be critical components of parasite biology. This is particularly valuable in *Leishmania*, whose divergent signalling landscape demands alternative strategies for functional characterization.

Beyond protein kinases, the functionally agnostic nature of the broad-spectrum probes means that they also identify other ATP-dependent enzymes. Notably amongst these was the substantial representation of metabolic kinases (e.g., pyruvate kinase, phosphoglycerate kinase) among the probe-enriched proteins. This finding has potential utility with certain parasite-specific metabolic enzymes being viable drug targets. For instance, the labelled *Leishmania* glycosomal phosphoenolpyruvate carboxykinase and homoserine kinase lack human orthologs, making them theoretically attractive targets. Similarly, nucleoside kinases like adenosine kinase are vital for salvaging host purines, essential for parasite DNA/RNA synthesis and growth (Boitz et al., 2012).

Our ABPP analysis revealed dynamic changes in the *Leishmania* profile that occur across the life cycle and between species. This represents a major advantage of the technique providing a real-time, native-state mapping of kinase activity without requiring any genetic modification of the organism. In general, procyclic promastigotes have a baseline set of active proteins geared towards growth, metacyclic promastigotes upregulate additional proteins in preparation for infectivity, and amastigotes activate a different set of proteins while turning off some promastigote signals. These patterns agree with prior transcriptomic and proteomic studies across *Leishmania* life-cycle transitions (Dillon et al., 2015; Fiebig et al., 2015; Inbar et al., 2017; Isern et al., 2025). This dynamic regulation emphasizes that simply knowing a gene is present is insufficient, one must assess when and where it is active. These insights demonstrate the power of ABPP in capturing stage-dependent biochemistry in parasites. Additionally, activity-based readouts have been explored for infectious-disease diagnostics (Keller et al., 2020; Chan, 2021). The fingerprint differences we observed between species open the possibility of using ABPP to aid *Leishmania* discrimination; however, any diagnostic application would require validation in clinical samples (with appropriate performance metrics).

This work reveals significant implications for drug discovery. Firstly, we provide a prioritised list of 48 functionally ligandable, active kinase targets (Supplementary Table S1). Many belong to enzyme classes heavily pursued in other diseases, potentially accelerating repurposing efforts (e.g., screening human kinase inhibitor libraries against *Leishmania*). Our findings can rationalize why certain broad-spectrum inhibitors like imatinib or sorafenib show anti-leishmanial activity (Cayla et al., 2022), likely hitting multiple active parasite kinases (e.g., CRKs, MAPKs), in a manner similar to ibrutinib. ABPP can directly aid target deconvolution: treating parasites with an inhibitor followed by ABPP can reveal which kinase labels disappear, pinpointing targets engaged by the compound. This addresses a major bottleneck in phenotypic screening. Secondly, ABPP allows assessment of compound selectivity within the parasite’s biological context. A critical challenge is ensuring inhibitors kill parasites via intended targets rather than unknown off targets (Isern et al., 2025). Profiling the kinome of drug-treated parasites, as demonstrated for clinical inhibitors in human cells (Patricelli et al., 2007), can map on- and off-target engagement, validating mechanism of action and guiding lead optimization. Thirdly, the ABPP approach is scalable and adaptable for various applications, including inhibitor screening or cellular imaging (Porta and Steel, 2023).

Methodologically, our study validates ABPP as a potent addition to *Leishmania* research tools. We demonstrated the utility of membrane-permeable ATP-directed probes in living parasites, capturing 48 kinases likely active under tested conditions. This was achieved with a very small set of probes and future iterations employing probes with expanded chemotypes can enhance target coverage and facilitate profiling under a broader range of physiological contexts, including in the disease critical intramacrophage amastigote. Furthermore, these probes were designed to engage ligandable ATP sites and, following affinity enrichment and TMT labelling, allow quantification of many low-abundance kinases (McAllister et al., 2013; Ray et al., 2019). Sensitivity is governed by probe accessibility and MS depth; very low-abundance or occluded sites may fall below detection. In this respect, it is acknowledged that while ABPP enriches for active kinases, it might potentially miss those with low reactivity, highly transient activity states, or ATP-pocket configurations not optimally recognised by the current probe designs. Notably, large, atypical kinases like TOR were absent from our datasets, emphasizing ABPP’s complementary role alongside genetics, phosphoproteomics, and structural analyses for achieving a holistic kinome map. Work towards these goals is in progress and will be reported in due course. Finally, to our knowledge, this is the first live-cell ABPP survey of the *Leishmania* kinome across species and life stages with competitive ABPP and protein-resolved TMT quantification, yielding a prioritised set of kinase targets for follow-up.

## 4 Conclusion

In conclusion, this study demonstrates the feasibility and significant value of activity-based chemoproteomic profiling for mapping *Leishmania’s* active kinome. By illuminating which parasite kinases are pharmacologically accessible in their active state, we lay crucial groundwork for next-generation therapies. These insights, together with ongoing medicinal chemistry efforts, accelerate the prospect of kinase-centred treatments for leishmaniasis, potentially yielding more effective, selective, and durable therapies against this debilitating disease. We identified kinases critical for parasite viability, providing robust validation for previously predicted targets and revealing novel candidates for therapeutic intervention. By highlighting stage- and species-specific kinase activities, we contribute leads for exploring parasite adaptation mechanisms. Importantly, our work further supports the use of ABPP as a versatile, functional proteomics platform capable of systematically identifying and prioritizing druggable enzyme targets. As antiparasitic drug discovery increasingly prioritizes validated functional targets, ABPP emerges as an indispensable tool, shifting paradigms from genomics-focused to functionally informed proteomic strategies. Ultimately, chemoproteomic-guided drug development, targeting enzymes essential to parasite survival and virulence, offers innovative solutions amidst emerging drug resistance and a critical need for novel therapeutic leads.

## 5 Materials and methods

### 5.1 General conditions

All solvents and reagents were purchased from commercial suppliers. NMR spectra were recorded on the following instruments: Bruker Neo 700 MHz spectrometer with operating frequencies of 699.73 MHz for ^1^H, 175.95 MHz for ^13^C, 658.41 MHz for ^19^F; Bruker Neo-400 spectrometer with operating frequencies of 400.20 MHz for ^1^H, 100.63 MHz for ^13^C, 376.57 MHz for ^19^F. Spectra were referenced relative to CDCl_3_ (δ_H_ 7.26 ppm, δ_C_ 77.16 ppm) or DMSO-d_6_ (δ_H_ 2.50 ppm, δ_C_ 39.52 ppm). Chemical shifts are reported in parts per million (ppm), coupling constants (*J*) in hertz (Hz) and multiplicity as singlet (s), doublet (d), triplet (t), quartet (q), multiplet (m), or a combination thereof. All ^1^H NMR and ^13^C NMR spectral assignments were made with the aid of ^1^H–^1^H COSY, ^1^H-^13^C HSQC and ^1^H-^13^C HMBC NMR experiments. Infra-red spectra were recorded on a PerkinElmer Frontier FTIR spectrometer equipped with a Specac Quest ATR accessory with extended range diamond puck. IR assignments are reported in wavenumbers (cm^-1^). Thin layer chromatography was performed using Merck F_254_ silica gel 60 aluminum sheets pre-coated with silica gel. High-resolution mass spectrometry (HRMS) and liquid chromatography mass spectrometry (LCMS) were recorded on a Waters TQD mass spectrometer ESI-LC water (0.1% formic acid): MeCN, flow rate 0.6 mL min^-1^ with a UPLC BEH C18 1.7 μm (2.1 mm × 50 mm) column. Melting points (M.p.) are measured using Fisher ScientificTM IA9000 melting point apparatus.

### 5.2 Synthesis of probe 1: (*R*)-1-(3-(4-(pent-4-yn-1-ylamino)-3-(4-phenoxyphenyl)-1*H*-pyrazolo[3,4-*d*]pyrimidin-1-yl)piperidin-1-yl)prop-2-en-1-one

To a stirred solution of ibrutinib (50 mg, 0.113 mmol, 1.0 eq.) in anhydrous dimethylformamide (DMF, 5 mL) was added potassium carbonate (47 mg, 0.340 mmol, 3.0 eq.) under an inert argon atmosphere. The resulting suspension was stirred at room temperature for 10 min before the addition of 5-chloropentyne (18 μL, 0.170 mmol, 1.5 eq.). The reaction mixture was then heated to 110 °C. Additional portion of 5-chloropentyne (12 μL, 0.113 mmol, 1.0 eq.) was added to the reaction mixture every 48 h. After a total of 6 days, the reaction was cooled to ambient temperature and diluted with water (10 mL). The resulting aqueous mixture was extracted with ethyl acetate (3 × 10 mL). The combined organic phases were sequentially washed with water (15 mL) and saturated aqueous sodium chloride solution (brine, 15 mL), dried over anhydrous magnesium sulfate (MgSO_4_), filtered, and the solvent was removed *in vacuo*. The resulting crude residue was purified by flash column chromatography on silica gel, eluting with a gradient of 0%–20% methanol in dichloromethane. The corresponding fractions were combined and concentrated to afford Probe 1 as a white solid (12 mg, 21% yield). M.p. 126 °C–129 °C. ν_max_ (ATR) 3305, 3295, 2940, 1667, 1615, 1525, 1490 cm^-1^. ^1^H NMR (700 MHz, CDCl_3_) δ 8.77 (s, 1H), 7.85 (d, *J* 7.4, 2H), 7.67 (t, *J* 8.2, 2H), 7.45 (d, *J* 7.3, 2H), 7.24–7.03 (m, 3H), 6.64–6.45 (m, 1H), 6.45–6.28 (m, 1H), 5.81–5.73 (m, 1H), 4.91–4.77 (m, 1H), 4.61–4.38 (m, 1H), 3.95–3.84 (m, 1H), 3.77–3.60 (m, 1H), 3.51–3.30 (m, 3H), 3.22–2.99 (m, 1H), 2.85–2.75 (m, 1H), 2.75–2.62 (m, 1H), 2.30 (t, *J* 2.0, 1H), 2.05–1.97 (m, 2H), 1.97–1.89 (m, 1H), 1.76–1.58 (m, 3H). ^13^C NMR (176 MHz, CDCl_3_) δ 165.8, 158.3, 158.1,157.9, 155.2, 154.7, 143.8, 130.3, 129.8, 129.2, 126.4, 123.5, 119.5, 119.2, 98.6, 85.0, 71.5, 53.8, 52.8, 50.0, 46.2, 45.9, 42.2, 30.3, 29.9, 26.6, 24.2, 21.0. HRMS (ES^+^) found [M + H]^+^ 507.2478; C_30_H_31_N_6_O_2_ requires *M* 507.2503.

### 5.3 Synthesis of *Int-I*: N-(pent-4-yn-1-yl)-1*H*-pyrazolo[3,4-*d*ay]pyrimidin-4-amine

In a round-bottom flask, 4-chloro-1*H*-pyrazolo[3,4-*d*]pyrimidine (500 mg, 3.25 mmol, 1.0 eq.) was dissolved in anhydrous DMF (10 mL). To this solution were added 1-aminopent-4-yne (471 μL, 4.88 mmol, 1.5 eq.) and triethylamine (TEA, 1.36 mL, 9.75 mmol, 3.0 eq.) under an inert argon atmosphere. The reaction mixture was heated to 70 °C and stirred at this temperature for 8 h. Upon completion, the reaction was allowed to cool to ambient temperature and was subsequently quenched by the addition of 1 M aqueous HCl (1.0 mL). The crude solution was filtered, evaporated, and purified directly by preparative reverse-phase liquid chromatography utilising a gradient of water and acetonitrile. Then, the solvent was removed under reduced pressure and high vacuum to afford the title compound (425 mg, 65% yield) as a colourless oil. ν_max_ (ATR) 3324, 3275, 2966, 1610, 1530, 1498 cm^-1^. ^1^H NMR (400 MHz, DMSO-d_6_) δ 9.23 (bs, 1H), 8.24 (s, 1H), 7.80 (s, 1H), 7.23 (bs, 1H), 3.07–2.93 (m, 2H), 3.01 (t, *J* 2.1, 1H), 2.04–1.94 (m, 2H), 1.51–1.42 (m, 2H). ^13^C NMR (101 MHz, DMSO-d_6_) δ 153.2, 152.1, 150.1, 129.6, 99.5, 82.1, 72.5, 41.0, 24.1, 21.0. HRMS (ES^+^) found [M + H]^+^ 202.1092; C_10_H_12_N_5_ requires *M* 202.1087.

### 5.4 Synthesis of *Int-II*: tert-butyl (*R*)-3-(4-(pent-4-yn-1-ylamino)-1*H*-pyrazolo[3,4-*d*]pyrimidin-1-yl)piperidine-1-carboxylate

An oven-dried round-bottom flask was charged with triphenylphosphine (TPP, 782 mg, 3.0 mmol, 2.0 eq.) and placed under an inert argon atmosphere. Anhydrous tetrahydrofuran (THF, 5 mL) was added, and the resulting solution was cooled to 0 °C. To this stirred solution, diisopropyl azodicarboxylate (DIAD, 530 μL, 2.7 mmol, 1.8 eq.) was added dropwise over 5 min. The mixture was stirred at 0 °C for an additional 15 min. Subsequently, a solution containing N-(pent-4-yn-1-yl)-1*H*-pyrazolo[3,4-*d*ay]pyrimidin-4-amine (*Int-I*) (300 mg, 1.5 mmol, 1.0 eq.) and (*S*)-tert-butyl 3-hydroxypiperidine-1-carboxylate (600 mg, 3.0 mmol, 2.0 eq.) in anhydrous THF (2 mL) was added to the reaction mixture. The cooling bath was removed, and the reaction was allowed to warm to ambient temperature, stirring for 16 h. Upon completion, the solvent was removed *in vacuo* and purified by flash column chromatography on silica gel (gradient: 0%–20% v/v methanol in dichloromethane) to afford the title compound as a white solid (321 mg, 56%). M.p. 103 °C–104 °C. ν_max_ (ATR) 3363, 3266, 2927, 1698, 1601, 1542, 1501 cm^-1^. ^1^H NMR (400 MHz, CDCl_3_) δ 8.29 (s, 1H), 7.55 (s, 1H), 4.80–4.74 (m, 1H), 4.44–4.20 (m, 2H), 3.81 (bs, 1H), 3.66–3.54 (m, 1H), 3.07–3.01 (m, 1H), 2.87–2.70 (m, 2H), 2.43–2.32 (m, 2H), 2.26 (t, *J* 2.3, 1H), 2.12–2.02 (m, 2H), 1.86–1.72 (m, 4H), 1.41 (s, 9H). ^13^C NMR (101 MHz, CDCl_3_) δ 157.3, 153.2, 152.1, 147.9, 129.6, 99.7, 84.1, 82.4, 72.5, 53.5, 41.7, 41.1, 30.8, 28.4, 27.2, 26.6, 22.1, 21.0. HRMS (ES^+^) found [M + H]^+^ 385.2329; C_20_H_29_N_6_O_2_ requires *M* 385.2347.

### 5.5 Synthesis of *Int-III*: (*R*)-N-(pent-4-yn-1-yl)-1-(piperidin-3-yl)-1*H*-pyrazolo[3,4-*d*]pyrimidin-4-amine

The Boc-protected *Int-II* intermediate (150 mg, 0.39 mmol) was dissolved in dry DCM (2 mL) and cooled to 0 °C. Trifluoroacetic acid (1 mL) was added in a single portion under an inert argon atmosphere. The resulting reaction mixture was warmed to room temperature and stirred for 1 h. The reaction progress was monitored by TLC and by LC-MS to confirm the complete consumption of the starting material. Upon completion, the volatiles were removed *in vacuo*. To ensure complete removal of residual acid, the crude residue was co-evaporated with toluene (2 × 5 mL). This afforded the desired amine as its TFA salt (161 mg, >95% by LC-MS), which appeared as a pale-yellow oil and was carried forward to the next synthetic step without any further purification.

### 5.6 Synthesis of probe 2: (*R*)-1-(3-(4-(pent-4-yn-1-ylamino)-1*H*-pyrazolo[3,4-*d*]pyrimidin-1-yl)piperidin-1-yl)prop-2-en-1-one

The crude amine *Int-III* trifluoroacetate salt (50 mg, 0.12 mmol, 1.0 eq.) was dissolved in anhydrous tetrahydrofuran (4 mL) in an oven-dried flask under an inert argon atmosphere. The solution was cooled to 0 °C. To this, TEA (175 μL, 1.25 mmol, 10.0 eq.) was then added and the mixture was stirred for 15 min at 0 °C. Subsequently, a solution of acryloyl chloride (15 μL, 0.18 mmol, 1.5 eq.) in anhydrous THF (1 mL) was added dropwise via syringe. The reaction was maintained at 0 °C and stirred until complete consumption of the starting amine (2 h). The reaction was quenched by the addition of saturated aqueous NaHCO_3_ solution (10 mL). The aqueous layer was separated and extracted with ethyl acetate (3 × 15 mL). The combined organic extracts were washed with water (15 mL), followed by brine solution (15 mL), then dried over anhydrous MgSO_4_, filtered, and the solvent was removed *in vacuo*. The resulting residue was purified by flash column chromatography on silica gel using a gradient of methanol in dichloromethane (from 0% to 10% v/v) and concentrated to yield Probe 2 as a white solid (16 mg, 37% over two steps). M.p. 89 °C–91 °C. ν_max_ (ATR) 3322, 2952, 1670, 1610, 1522, 1490 cm^-1^. ^1^H NMR (400 MHz, CDCl_3_) δ 8.12 (s, 1H), 7.69 (s, 1H), 6.43–6.35 (m, 1H), 6.26–6.18 (m, 1H), 5.77–5.66 (m, 1H), 4.77–4.65 (m, 1H), 4.63–4.51 (m, 2H), 4.31–4.22 (m, 1H), 3.88–3.79 (m, 1H), 3.67–3.39 (m, 3H), 3.12–3.04 (m, 1H), 2.81–2.74 (m, 1H), 2.74–2.59 (m, 1H), 2.32 (t, *J* 2.2, 1H), 2.22–2.07 (m, 2H), 1.87–1.64 (m, 3H). ^13^C NMR (101 MHz, CDCl_3_) δ 164.9, 156.7, 154.7, 154.5, 132.0, 130.8, 126.3, 101.9, 85.1, 71.4, 65.5, 46.0, 45.4, 42.8, 28.3, 26.7, 21.1, 20.5. HRMS (ES^+^) found [M + H]^+^ 339.1955; C_18_H_23_N_6_O requires *M* 339.1928.

### 5.7 Synthesis of probe 3: (*R*)-4-(3-(4-(pent-4-yn-1-ylamino)-1*H*-pyrazolo[3,4-*d*]pyrimidin-1-yl)piperidine-1-carbonyl)benzenesulfonyl fluoride

The crude amine *Int-III* trifluoroacetate salt (50 mg, 0.12 mmol, 1.0 eq.) was dissolved in anhydrous tetrahydrofuran (4 mL) in an oven-dried flask under an inert argon atmosphere. The solution was cooled to 0 °C. To this, TEA (175 μL, 1.25 mmol, 10.0 eq.) was then added and the mixture was stirred for 15 min at 0 °C. Subsequently, a solution of 4-(fluorosulfonyl) benzoyl chloride (40 mg, 0.18 mmol, 1.5 eq.) in anhydrous THF (1 mL) was added dropwise via syringe. The reaction was warmed to room temperature and stirred until complete consumption of the starting amine (2 h). The reaction was quenched by the addition of saturated aqueous NaHCO_3_ solution (10 mL). The aqueous layer was separated and extracted with ethyl acetate (3 × 15 mL). The combined organic extracts were washed with water (15 mL), followed by brine solution (15 mL), then dried over anhydrous MgSO_4_, filtered, and the solvent was removed *in vacuo*. The resulting residue was purified by flash column chromatography on silica gel using a gradient of methanol in dichloromethane (from 0% to 10% v/v) and concentrated to yield Probe 3 as a white solid (46 mg, 82% over two steps). M.p. 126 °C–127 °C. ν_max_ (ATR) 3305, 3113, 2966, 1682, 1620, 1490, 1404, 1206 cm^-1^. ^1^H NMR (700 MHz, CDCl_3_) δ 8.26 (s, 1H), 8.12 (d, *J* 8.2, 2H), 7.75 (d, *J* 8.1, 2H), 7.55 (s, 1H), 4.67–4.58 (m, 1H), 4.33–4.18 (m, 2H), 3.67–3.42 (m, 2H), 3.22–3.17 (m, 1H), 2.94–2.84 (m, 1H), 2.73–2.66 (m, 1H), 2.58–2.52 (m, 1H), 2.43–2.36 (m, 1H), 2.22–2.18 (m, 1H), 2.07–1.87 (m, 3H), 1.63–1.51 (m, 3H). ^13^C NMR (176 MHz, CDCl_3_) δ 165.4, 161.6, 160.9, 160.4, 157.6, 155.1, 152.4, 144.9, 135.0, 129.6, 129.2, 114.7, 114.3, 114.1, 99.5, 82.1, 72.7, 55.9, 41.5, 41.1, 31.4, 27.2, 21.8, 21.1. ^19^F NMR (658 MHz, CDCl_3_) δ 65.0. HRMS (ES^+^) found [M + H]^+^ 471.1584; C_22_H_24_N_6_O_3_SF requires *M* 471.1609.

### 5.8 *Leishmania* cell cultures

*L. major* (MHOM/IL/81/Friedlin; WT), *L. mexicana* (MHOM/SA/85/JISH118; WT), and *L. amazonensis* (MHOM/BR/75/Josefa; WT) promastigotes were maintained at 26 °C in Schneider’s insect medium (pH 7), supplemented with 15% heat-inactivated fetal bovine serum (FBS), 100 μg mL^−1^ streptomycin, and 100 IU mL^−1^ penicillin. *L. mexicana* axenic amastigotes were maintained at 32 °C in Schneider’s insect medium (pH 5.5), supplemented with 15% heat-inactivated fetal bovine serum (FBS), 100 μg mL^−1^ streptomycin, and 100 IU mL^−1^ penicillin.

### 5.9 *Leishmania mexicana* cell culture for life cycle

*L. mexicana* promastigotes were maintained at 26 °C in Schneider’s *Drosophila* medium supplemented with 15% FBS and 1% Penicillin/Streptomycin. *Leishmania* promastigotes in the late log phase were then transferred into Schneider’s Insect medium supplemented with 20% FBS (pH 7) at a density of 5 × 10^5^ parasites per mL. Metacyclic promastigotes were retrieved after 6 days of incubation at 26 °C.

*L. mexicana* axenic amastigotes were maintained at 32 °C in Schneider’s insect medium (pH 5.5), supplemented with 15% heat-inactivated fetal bovine serum (FBS), 100 μg mL^−1^ streptomycin, and 100 IU mL^−1^ penicillin.

### 5.10 Anti-parasitic assay

*L. mexicana* (2 × 10^6^ mL^−1^) promastigotes and axenic amastigotes were incubated in sterile 96-well plates with compounds in triplicate (amphotericin B was used as a positive control and untreated parasites with DMSO, as a negative control) at 26 °C (or 32 °C for axenic amastigotes) for 72 h. Resazurin solution (10 μL) was then added and the plate, incubated at 26 °C for 4 h prior to measurement using a fluorescence plate reader (Ex555/Em585). At least three independent experiments were performed for each molecule with all samples in triplicates. EC_50_ values were calculated using sigmoidal regression analysis (GraphPad Prism).

### 5.11 Protein extraction from *Leishmania* parasites

Parasites were harvested by centrifugation (1000 × g, 5 min, 4 °C), washed three times with cold Dulbecco’s phosphate-buffered saline (PBS, pH 7.4), and lysed with lysis buffer (25 mM Tris–HCl (pH 7.4), 150 mM NaCl, 1% Triton X-100, and 5% glycerol). For lysate labelling, cysteine protease inhibitor E−64 (10 mM) was added, while for whole-parasite labelling, a cOmplete Mini, EDTA-free protease inhibitor cocktail (Roche 1183170001) was used. The resulting lysates were centrifuged (13,000 × g, 10 min, 4 °C) to remove insoluble material. Protein concentration in each sample was determined using Pierce Rapid Gold BCA Protein Assay Kit (ThermoFisher) following the manufacturers’ protocol, and homogenates were adjusted to the stated protein concentration (1–1.5 mg/mL).

### 5.12 Live parasites ABP labelling

Parasites were incubated with the stated probes (ABPs) (10 μM) for 2 h at 26 °C (at 32 °C for *L. mexicana* axenic amastigotes), followed by lysis using the previously described procedure. The lysis buffer included cOmplete Mini, EDTA-free protease inhibitor cocktail (Roche 1,183,170,001).

### 5.13 Biorthogonal Cu-Catalysed cycloaddition (CuAAC)

Rhodamine-N_3_/biotin-N_3_ (50 μM) or Biotin-N_3_ (50 μM), CuSO_4_ (1 mM), TBTA (0.1 mM), and sodium ascorbate (1 mM) were added to the ABP-labelled tissue homogenate (1–1.5 mg/mL). The mixture was incubated at room temperature for 1 h, with periodic mixing. Upon completion, the reaction was stopped by adding 4X LDS sample buffer (200 mM Tris–HCl, pH 6.8, 400 mM DTT, 8% LDS, 0.04% bromophenol blue, and 40% glycerol) and incubated at 95 °C for 5 min. Proteins were resolved on a 12.5% (w/v) SDS-PAGE, and fluorescent bands were detected using a Typhoon 9400 Variable Mode Imager. Emission filter: 580 BP 30 Cy3, TAMRA, AlexaFluor546; Laser: green (532 nm). Gels were subsequently stained with Coomassie Brilliant Blue R-250 (CBB), and the images were documented. ABPs and reporter tag stocks were prepared in DMSO (Sigma Aldrich).

### 5.14 Competitive ABPP study

To determine the sensitivity of the detected kinases towards a well-known inhibitor of kinases, we performed the competitive ABPP labelling with ibrutinib. Competitive ibrutinib labelling was conducted with parasites by pre-incubation with different concentrations of inhibitor (0.01–100 μM) or DMSO (as a control) for 4 h at 26 °C. After incubation, 10 μM of probes were added into each experiment and kept for 2 h at 26 °C, followed by lysis using the previously described procedure. The lysis buffer included cOmplete Mini, EDTA-free protease inhibitor cocktail (Roche 1183170001). After CuAAC with Rh-N_3_, separation and detection of ABPP labelled proteins were done as described above. Fluorescence profiles and peak volume quantitation for labelled proteins were generated using the instrument’s ImageQuant TL v2005 software.

### 5.15 Protein precipitation

After biotin attachment by biorthogonal click chemistry (CuAAC), proteins were precipitated by the addition of nine volumes of ice-cold MeOH and stored overnight at −80 °C. Subsequently, lysates were centrifuge at 10,000 x g for 10 min at 4 °C and washed twice with ice-cold MeOH to remove unreacted ABPs, biotin-N_3_, and click chemistry reagents. Finally, pellets were air-dried for 30 min.

### 5.16 Affinity enrichment

Precipitated proteins were redissolved in a minimal volume of 2% SDS (in PBS) and diluted to a final concentration of 0.1% SDS. NeutrAvidin-Agarose beads (50 µL per sample), freshly washed three times with four volumes of 0.1% SDS (in PBS), were added to each of the samples, and the mixture was rotated on an end-over-end rotating shaker for 2 h at room temperature. The beads were then washed three times with 0.5% SDS in PBS, three times with 6M urea in PBS, three times with PBS, and once with 50 mM TEAB buffer. Each washing was performed with 500 μL of the washing solution, and centrifugation was carried out at 1,500 × g for 2 min at room temperature.

### 5.17 On-bead reduction, alkylation, and tryptic digestion

Washed beads from the previous affinity enrichment step were treated with 200 µL 10 mM TCEP in 50 mM TEAB buffer for 45 min at 30 °C. After this period, the samples were washed by the addition of 400 µL of 50 mM TEAB buffer, centrifuged at 1500 *g* for 2 min, and the supernatant removed. Subsequently, the beads were resuspended in 200 µL of 15 mM a-iodoacetamide in 50 mM TEAB buffer and incubated in the dark for 45 min. The beads were again washed with 50 mM TEAB buffer, resuspended in 200 µL of fresh 100 mM TEAB buffer, and treated with 4 µg of sequencing-grade modified trypsin at 37 °C for 16 h. The samples were centrifuged at 5000 *g* for 5 min, and the supernatant collected. The remaining beads were washed twice with 50 μL of 50% ACN containing 0.1% FA, centrifuged at 1500 *g* for 2 min, and the supernatants were mixed. The collected tryptic peptides were acidified to pH = 3 using formic acid and evaporated to dryness. The peptides were then redissolved in 0.1% (v/v) formic acid solution in water and desalted using Pierce™ Peptide Desalting Spin Columns (Thermo Scientific; CN: 89851) following manufacturer’s instructions. The remaining peptides were finally evaporated to complete dryness under a vacuum.

### 5.18 TMT labelling

Dried and desalted tryptic peptides were resuspended in 100 μL of 100 mM TEAB. Subsequently, 41 μL of the previously equilibrated TMT10plex Mass Tag Labelling reagents (Thermo Scientific, CN: 90110) were added to each sample and incubated for 1 h at room temperature, followed by the addition of 8 μL of 5% hydroxylamine to each sample and incubated for a further 15 min to quench the reaction. Upon completion, equal amounts of each sample were combined in a new microcentrifuge tube and evaporated to dryness. Finally, the peptides were desalted using Pierce™ Peptide Desalting Spin Columns (Thermo Scientific; CN: 89851) following manufacturer’s instructions and evaporated to dryness.

### 5.19 Nano LC-MS/MS data acquisition

The LC-MS/MS analyses of TMT-labelled peptides were performed by the Proteomics Core Facility of The Institute of Cancer Research, London, UK, on an Orbitrap Ascent Mass Spectrometer (Thermo Fisher Scientific) coupled with a Thermo Scientific Ultimate 3000 RSLCnano UHPLC system (Thermo Fisher Scientific). Desalted and TMT-labelled tryptic peptides dissolved in 0.1% formic acid (FA) were first loaded onto an Acclaim PepMap 100 C18 trap column (5 µm particle size, 100 µm ID X 20 mm, TF164564) heated to 45 °C using 0.1% FA/H_2_O with a flow rate of 10 μL/min, then separated on a Waters nanoEase M/Z Peptide BEH C18 Column (1.7 µm particle size, 130Å, 75 µm ID X 250 mm, 186008795) with a 5%–35% ACN gradient in 0.1% FA over 150 min at a flow rate of 300 nL/min. The full MS spectra (*m/z* 375 to 1,500) were acquired in Orbitrap at 120,000 resolutions with an AGC target value of 4e^5^ for a maximum injection time of 251 m. High-resolution HCD MS^2^ spectra were generated in positive ion mode using a normalised collision energy of 38% within a 0.7 m*/z* isolation window using quadrupole isolation. The AGC target value was set to 10e^4^, and the dynamic exclusion was set to 45 s. The MS^2^ spectra were acquired in Orbitrap with a maximum injection time of 80 m at a resolution of 45,000 with an instrument determined scan range beginning at *m/z* 100. To ensure quality peptide fragmentation a number of filters were utilised, including peptide monoisotopic precursor selection, minimum intensity exclusion of 50,000 and exclusion of precursor ions with unassigned charge state as well as charge state of +1 or superior to +7 from fragmentation selection. To prevent repeat sampling, a dynamic exclusion with exclusion counts of 1, exclusion duration of 45 s, mass tolerance window of ±7 ppm and isotope exclusion were used.

### 5.20 Proteomics MS data processing

All raw LC-MS/MS data were processed using MaxQuant software (Cox and Mann, 2008) version 1.6.3.4 with integrated Andromeda database search engine (Cox et al., 2011). The MS/MS spectra were queried against *L. mexicana* sequences from UniProt KB (8,559 sequences). The following search parameters were used: reporter ion MS^2^ with multiplicity 10-plex TMT, trypsin digestion with maximum two missed cleavages, carbamidomethylation of cysteine as a fixed modification, oxidation of methionine, acetylation of protein N-termini as variable modifications, minimum peptide length of 6, a maximum number of modifications per peptide set at 5, and protein false discovery rate (FDR) 0.01. Appropriate correction factors for the individual TMT channels, accounting for both lysine side-chain labelling and peptide N-terminal labelling, as per the TMT-10plex kits used (Thermo Fisher Scientific, CN: 90110) were configured into the database search. The proteinGroups.txt files from the MaxQuant search outputs were processed using Perseus software version 1.6.10.50 (Tyanova et al., 2016). Sequences only identified by site, reverse sequences, and potential contaminants were filtered out. The reporter intensities were transformed to log_2_ scale. A modified *t-*test with permutation-based FDR statistics (250 permutations) was applied to compare the probe treatments versus control groups.

## Data Availability

The data presented in the study are deposited in the ProteomeXchange Consortium via the PRIDE partner repository, accession number PXD066933.
