# Supplementary material for: Activity-based chemoproteomic profiling reveals the active kinome of Leishmania
Source: Front Pharmacol. 2026 Jan 6;16:1687590. doi: 10.3389/fphar.2025.1687590 (PMC12816314; doi:10.3389/fphar.2025.1687590)
Supplement: Supplementary file 1 [file DataSheet1.pdf]

Supplementary Material

Activity-Based Chemoproteomic Profiling Reveals the Active Kinome of *Leishmania*

Exequiel O. J. Porta,[1,#,*] Karunakaran Kalesh,[2,3,*] Patrick G. Steel.[1,*]

[1] Department of Chemistry, Durham University, Durham, United Kingdom

[2] School of Health and Life Sciences, Teesside University, Middlesbrough, United Kingdom

[3] National Horizons Centre, Darlington, United Kingdom

[#] Current address: UCL School of Pharmacy, University College London, United Kingdom

*** Correspondence:**
Exequiel O. J. Porta, e.porta@ucl.ac.uk; Karunakaran Kalesh, K.Karunakaran@tees.ac.uk; Patrick G. Steel, p.g.steel@durham.ac.uk

Contents

1 Supplementary Figures and Tables 2

1.1 Supplementary Figures 1–5 2

1.2 Supplementary Table 7

2 Appendix 11

1 Supplementary Figures and Tables

1.1 Supplementary Figures

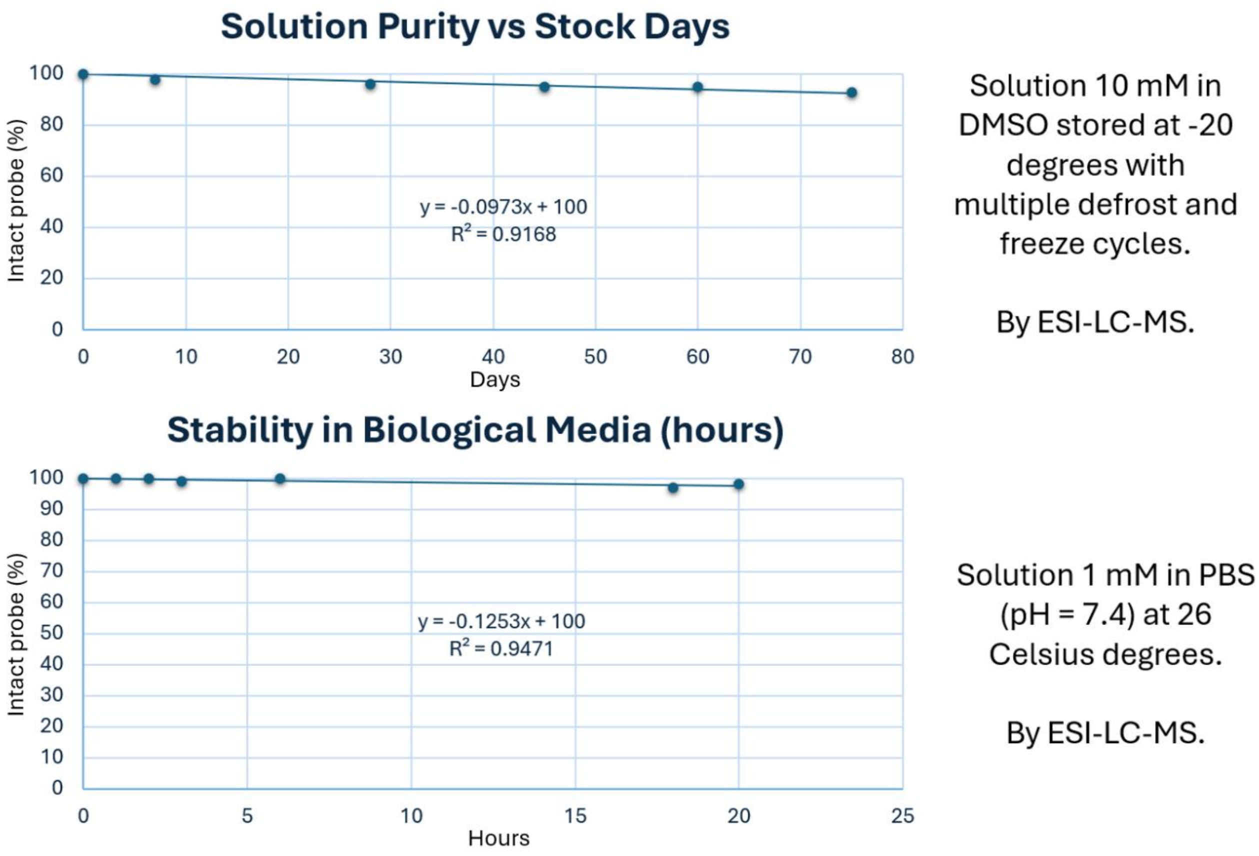

Supplementary Figure 1. Stability of the probe in stock solution and physiological buffer. (**Top**) Stability of a 10 mM stock solution of the probe in DMSO stored at −20 ^{0}C over 75 days, subjected to multiple freeze-thaw cycles. (**Bottom**) Stability of a 1 mM solution of the probe in phosphate-buffered saline (PBS, pH 7.4) at 26 ^{0}C over 20 hours. In both experiments, the percentage of remaining intact probe was quantified by ESI-LC-MS.

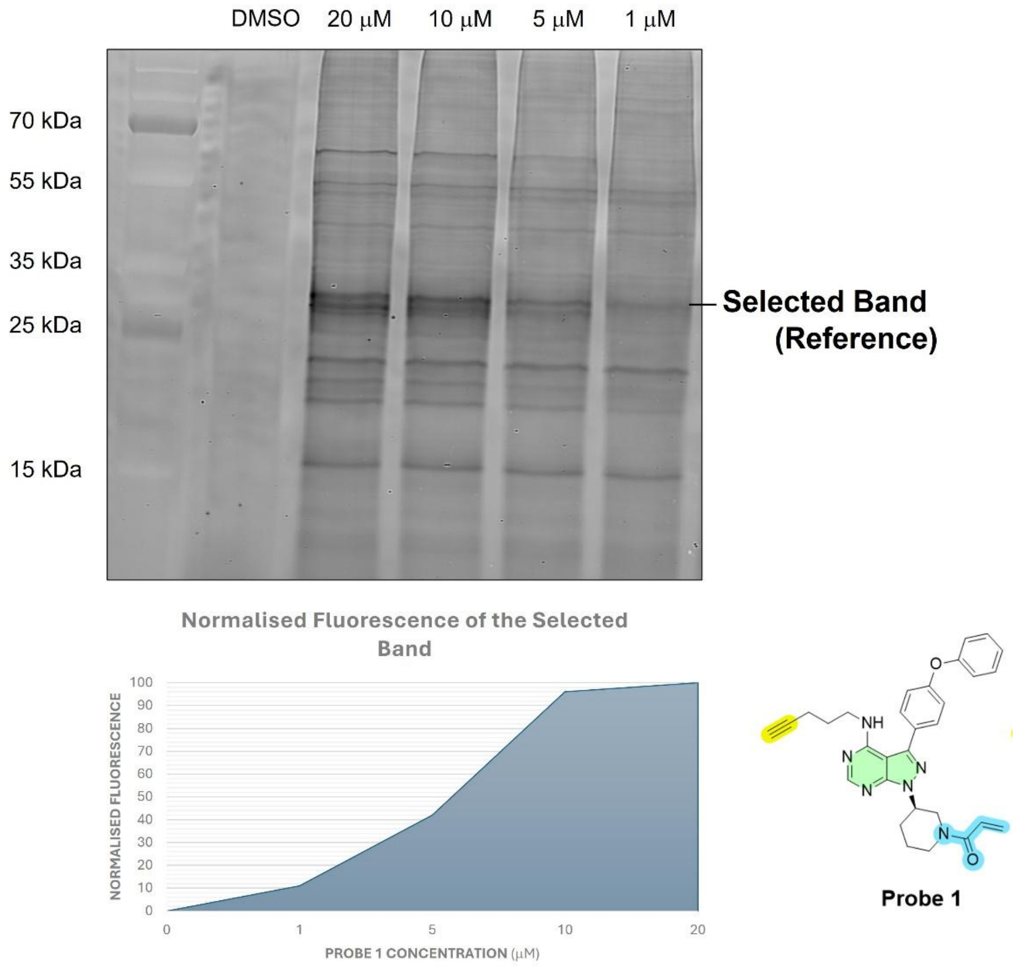

Supplementary Figure 2. Dose–response of Probe 1 labelling in *L. mexicana* promastigotes. Intact parasites were incubated with Probe **1** (1, 5, 10, 20 µM) or DMSO for 2 h, lysed, subjected to CuAAC with a fluorescent TAMRA azide, and resolved by SDS–PAGE (final protein concentration: 1 mg/mL). Equal protein was loaded, and images were acquired with identical settings (Typhoon laser scanner, 532 nm excitation/580 nm emission; ImageQuant TL, normal sensitivity, PMT 500–600 V). The gel (**top**) shows a concentration-dependent increase in probe-derived bands with a plateau at ~10–20 µM. The plot (**bottom**) quantifies the fluorescence of the indicated reference band, normalised to the maximum signal, and supports the selection of 10 µM for subsequent experiments.

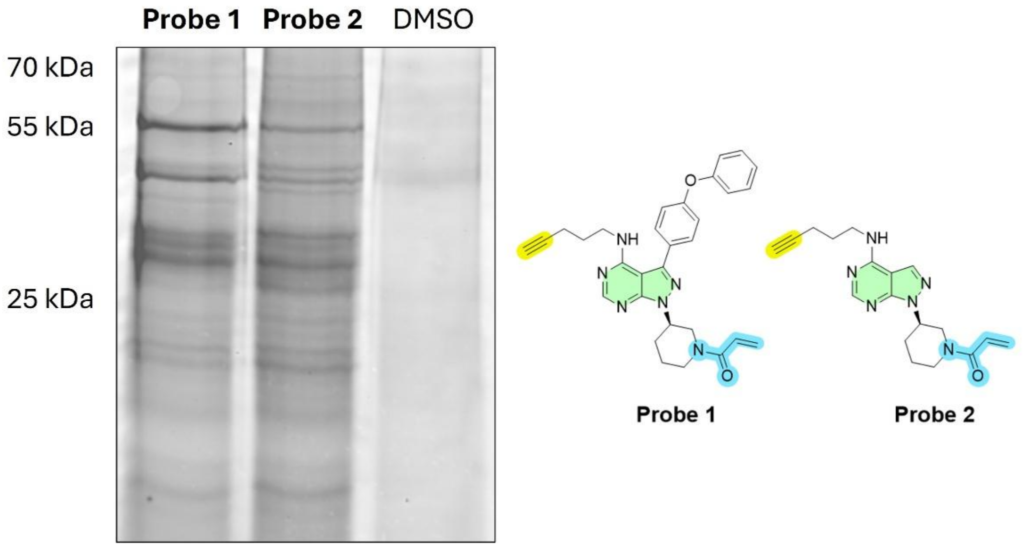

Supplementary Figure 3. In-gel ABPP comparison of acrylamide-based probes (Probe 1 and Probe 2) versus DMSO. In-gel fluorescence analysis (with emission detected at 580 nm) of *L. mexicana* at a final protein concentration of 1 mg/mL revealed an active protein fingerprint upon treatment with our 10 µM probes (2 h). Lane 1: Probe **1**; Lane 2: Probe **2**; Lane 3: DMSO (Control). Typhoon laser scanner (excitation 532 nm, emission 580 nm) and Image Quant TL software (normal sensitivity and PMT 500 or 600 V; GE Healthcare Life Sciences).

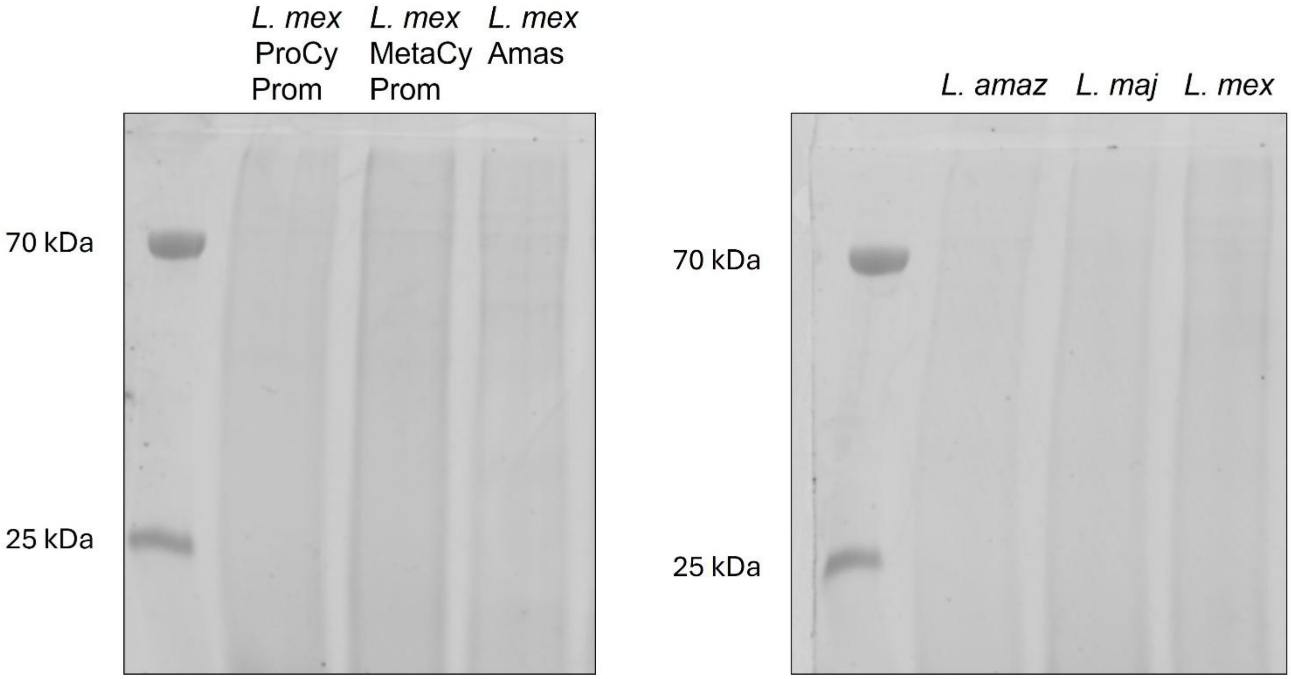

Supplementary Figure 4. No-probe (DMSO) controls. Left: DMSO-treated *L. mexicana* life stages processed identically to probe-treated samples (CuAAC, in-gel fluorescence). Final protein concentration: 1 mg/mL. **Right:** DMSO-treated *Leishmania* species (*L. amazonensis*, *L. major*, *L. mexicana*) processed identically to probe-treated samples (CuAAC, in-gel fluorescence). In both panels, no probe-derived fluorescent bands are detected, indicating minimal CuAAC/background signal.

```
Rabbit        --------------------MSKS--HSEAGSAFIQTQQLHAAMADTFLEHMCRLDIDSA    38
Leishmania    MGSSHHHHHHSSGLVPRGSHMGSSHHHHHHSSGLVPRG-----SHMSQLAHNLTLSIFDP    55
                                  *..*  * . .*.::           : * *   *.* .

Rabbit        PITARNTGIICTIGPASRSVETLKEMIKSGMNVARMNFSHGTHEYHAETIKNVRTATESF    98
Leishmania    VANYRAARIICTIGPSTQSVEALKGLIQSGMSVARMNFSHGSHEYHQTTINNVRQAAAEL    115
                . * : *******:::***:** :*:***.*********:****  **:*** *: .:

Rabbit        ASDPILYRPVAVALDTKGPEIRTGLIKGSGTAEVELKKGATLKITLDNAYMEKCDENILW    158
Leishmania    G------VNIAIALDTKGPEIRTGQFVGGD---AVMERGATCYVTTDPAFADKGTKDKFY    166
              .        :*:************ : *..    . :::***  :* * *: :*  :: ::

Rabbit        LDYKNICKVVDVGSKVYVDDGLISLQVKQKGPD-FLVTEVENGGFLGSKKGVNLPGAAVD    217
Leishmania    IDYQNLSKVVRPGNYIYIDDGILILQVQSHEDEQTLECTVTNSHTISDRRGVNLPGCDVD    226
              :**:*:.***  *. :*:***:: ***:.:  :  *   * *.  :..::******. **

Rabbit        LPAVSEKDIQDLKFGVEQDVDMVFASFIRKAADVHEVRKILGEKGKNIKIISKIENHEGV    277
Leishmania    LPAVSAKDRVDLQFGVEQGVDMIFASFIRSAEQVGDVRKALGPKGRDIMIICKIENHQGV    286
              ***** **  **:*****.***:******.* :* :*** ** **::* **.*****:**

Rabbit        RRFDEILEASDGIMVARGDLGIEIPAEKVFLAQKMIIGRCNRAGKPVICATQMLESMIKK    337
Leishmania    QNIDSIIEESDGIMVARGDLGVEIPAEKVVVAQKILISKCNVAGKPVICATQMLESMTYN    346
              :.:*.*:* ************:*******.:***::*.:** ***************  :

Rabbit        PRPTRAEGSDVANAVLDGADCIMLSGETAKGDYPLEAVRMQHLIAREAEAAMFHRKLFEE    397
Leishmania    PRPTRAEVSDVANAVFNGADCVMLSGETAKGKYPNEVVQYMARICLEAQSALNEYVFFNS    406
              ******* *******::****:*********.** *.*:    *. **::*: .  :*:.

Rabbit        LARSSSHSTDLMEAMAMGSVEASYKCLAAALIVLTESGRSAHQVARYRPRAPIIAVTRNH    457
Leishmania    IKKLQHIPMSADEAVCSSAVNSVYETKAKAMVVLSNTGRSARLVAKYRPNCPIVCVTTRL    466
              : : .    .  **:. .:*:: *:  * *::**:::****: **:***..**:.** .

Rabbit        QTARQAHLYRGIFPVVC-KDPVQEAWAEDVDLRVNLAMNVGKARGFFKKGDVVIVLTGWR    516
Leishmania    QTCRQLNITQGVESVFFDADKLGHDWGKEH--RVAAGVEFAKSKGYVQTGDYCVVIHADH    524
              **.** :: :*:  *.   * : . *.::   **  .::..*::*:.:.**  :*: . :

Rabbit        PGSGFTNTMRVVPVP 531
Leishmania    KVKGYANQTRILLVE 539
                .*::*  *:: *
```

Supplementary Figure 5. Multiple sequence alignment of pyruvate kinase from rabbit muscle (KPYM_RABIT; UniProt P11974) and *Leishmania mexicana* (KPYK_LEIME; UniProt Q27686), generated with Clustal Omega v1.2.4 (EBI).[1] The proteins share ~49% identity and ~64% similarity across the full length (531 vs 539 aa). Clustal notation: "*" identical, ":" conserved, "." semi-conserved substitutions. The extensive conservation supports the use of rabbit PYK as a surrogate model for probe-validation experiments.

[1] Madeira, F., Madhusoodanan, N., Lee, J., Eusebi, A., Niewielska, A., Tivey, A.R.N., Lopez, R., Butcher, S. (2024). The EMBL-EBI Job Dispatcher sequence analysis tools framework in 2024. *Nucleic Acids Res.* **52**(W1):W521-W525. doi: 10.1093/nar/gkae241.

1.2 Supplementary Table

Supplementary Table 1. Curated list of the 48 kinases identified in *L. mexicana* promastigotes through treatment with all probes (10 µM), following TMT LC-MS/MS analysis.

Code ID	Name	Type	Essentiality[(a)]	Human Orthologous	Mass (kDa)	Captured by Probe [(b)]
**E8NHS0**	Mitogen-activated protein kinase	PK	Non-essential	No	41,1	3
**E9AV92**	Cell division protein kinase 2 homolog CRK1	PK	Essential	Yes	34,5	3
**E9ARG4**	Putative glycogen synthase kinase 3 beta	PK	Essential	Yes	40,7	1, 2 & 3
**E9AS98**	Putative mitogen activated protein kinase	PK	Essential	Yes	41,5	3
**E9ALH3**	ATP-dependent 6-phosphofructokinase	Metabolic	Essential	Yes	54,1	1, 2 & 3
**E9AM28**	Cofilin-like protein	PK	Non-essential	Yes	15,7	3
**E9B2B7**	Non-specific serine/threonine protein kinase	PK	Non-essential	No	48,4	3
**E9B4Z4**	Mitogen-activated protein kinase kinase (MEK1)	PK	Non-essential	Yes	42,9	3
**E9B6S9**	Protein kinase A catalytic subunit isoform	PK	Essential	Yes	42,1	3
**E9AK77**	Putative adenylate kinase	Metabolic	Essential	Yes	22,8	1, 2 & 3
**E9ALM1**	Non-specific serine/threonine protein kinase	PK	Non-essential	Yes	88,4	1, 2 & 3

**E9ARP5**	Protein kinase A catalytic	PK	Non-essential	Yes	38,2	3
**E9AWZ0**	Uncharacterised protein (Npk1-related protein kinase-like)	PK	Non-essential	No	63,9	3
**E9AYU4**	Ribokinase	Metabolic	Essential	Yes	35,3	3
**E9B0Q4**	Putative Pyridoxal kinase	Metabolic	Essential	Yes	33,1	3
**E9B182**	Putative homoserine kinase	Metabolic	Essential	No	36,2	1, 2 & 3
**E9B2V8**	Putative serine/threonine kinase	PK	Non-essential	No	71,1	3
**E9B3U3**	Putative guanylate kinase	Metabolic	Essential	Yes	23,0	2 & 3
**E9B5Y5**	Protein kinase domain-containing protein	PK	Non-essential	No	54,6	1, 2 & 3
**E9B6I7**	Putative glycerol kinase, glycosomal	Metabolic	Non-essential	Yes	55,8	1, 2 & 3
**E9AN28**	Putative mitogen-activated protein kinase	PK	Non-essential	Yes	46,3	3
**E9AP68**	Putative mitogen-activated protein kinase kinase	PK	Non-essential	Yes	120,9	3
**E9ASJ2**	Mitogen-activated protein kinase	PK	Non-essential	No	50,6	1, 2 & 3
**E9AUF0**	Phosphoglycerate kinase	Metabolic	Essential	Yes	51,6	2
**E9ANP4**	Pyruvate, phosphate dikinase	Metabolic	Non-essential	Yes	101,0	3

**E9AZ83**	Glycosomal phosphoenolpyruvate carboxykinase	Metabolic	Essential	No	58,4	3
**E9ALG7**	Putative serine/threonine-protein kinase	PK	Non-essential	No	56,2	3
**E9ARW6**	Uncharacterised protein	PK	Non-essential	No	30,9	3
**E9B376**	Nucleoside diphosphate kinase	Metabolic	Non-essential	Yes	16,7	3
**E9AXW8**	Putative serine/threonine-protein kinase	PK	Essential	No	44,5	3
**E9B0L7**	Putative adenosine kinase	Metabolic	Non-essential	Yes	37,2	1, 2 & 3
**E9B0K7**	Putative rac serine-threonine kinase	PK	Non-essential	Yes	57,6	3
**E9APA2**	Protein kinase domain-containing protein	PK	Non-essential	No	64,5	3
**E9B5P1**	Pyruvate kinase	Metabolic	Essential	Yes	54,0	1, 2 & 3
**E9AN59**	Mitogen-activated protein kinase	PK	Non-essential	No	43,7	1, 2 & 3
**E9AVA9**	Putative adenylate kinase	Metabolic	Essential	Yes	29,9	1, 2 & 3
**E9AYV7**	Protein kinase domain-containing protein (CRK6)	PK	Non-essential	No	37,4	3
**E9ASS2**	Non-specific serine/threonine protein kinase	PK	Non-essential	No	56,5	1, 2 & 3
**E9B5Y1**	Putative casein kinase	PK	Essential	Yes	39,9	3

**E9B6F3**	Galactokinase-like protein	Metabolic	Non-essential	Yes	53,3	1, 2 & 3
**E9ALA7**	Putative serine/threonine kinase	PK	Non-essential	No	33,6	1, 2 & 3
**E9AQ75**	Uncharacterised protein (BIKE)	PK	Non-essential	No	72,5	2 & 3
**E9ARW5**	PKAc3 - AKB1	PK	Non-essential	No	67,9	1, 2 & 3
**E9AU44**	Uncharacterised protein (ABC1)	Atypical PK	Essential	No	60,8	1, 2 & 3
**E9B1B3**	Phosphoglycerate kinase	Metabolic	Non-essential	No	57,7	3
**E9B296**	Putative serine/threonine-protein kinase	PK	Essential	No	49,8	3
**E9ALJ2**	Mitogen-activated protein kinase kinase	PK	Essential	Yes	41,5	3
**E8NHN2**	Activated C kinase protein	PK	Essential	No	34.4	1, 2 & 3

(a) Essentiality was assessed based on genetic ablation data (Baker et al., 2021)[2] for protein kinases, and through annotations from the TDR Targets database for metabolic kinases (Landaburu et al., 2019).[3]

(b) Significant enrichment was defined as $q < 0.05$ (permutation-based FDR) together with a stricter effect-size threshold of $\Delta\log_2FC \geq 1$ (≥2-fold) for the pairwise contrasts (Probe **3** vs Probe **1**, Probe **3** vs Probe **2**, Probe **2** vs Probe **1**). $\Delta\log_2FC$ (Probe X, Probe Y) = $\log_2FC$ (Probe X vs DMSO) – $\log_2FC$ (Probe Y vs DMSO) (e.g., Probe 3 − Probe 2).

[2] Baker, N., Catta-Preta, C. M. C., Neish, R., Sadlova, J., Powell, B., Alves-Ferreira, E. V. C., et al. (2021). Systematic functional analysis of Leishmania protein kinases identifies regulators of differentiation or survival. *Nature Communications* **12**:1244. doi: 10.1038/s41467-021-21360-8.

[3] Landaburu, L. U., Berenstein, A., Videla, S., Maru, P., Shanmugam, D., Chernomoretz, A., et al. (2019). TDR Targets 6: Driving drug discovery for human pathogens through intensive chemogenomic data integration. *Nucleic Acids Research* **48**:D1010-D1020. doi: 10.1093/nar/gkz999.

2 Appendix

2.1 Int-I

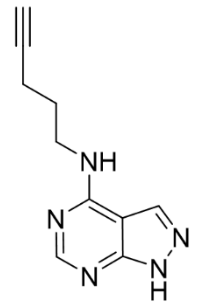

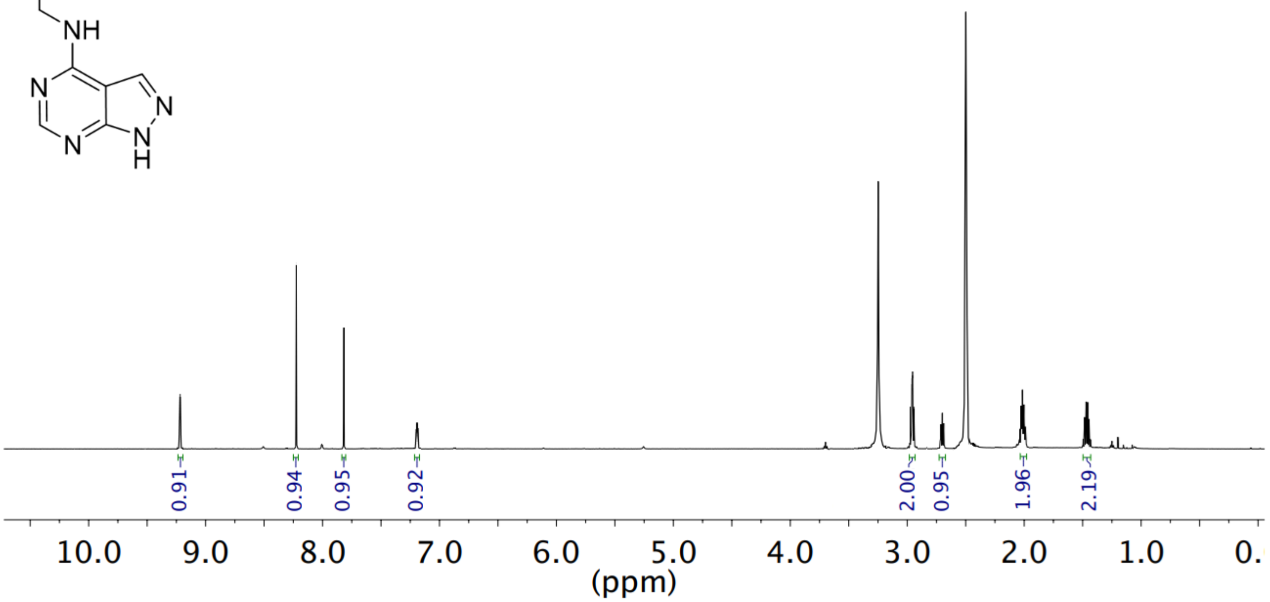

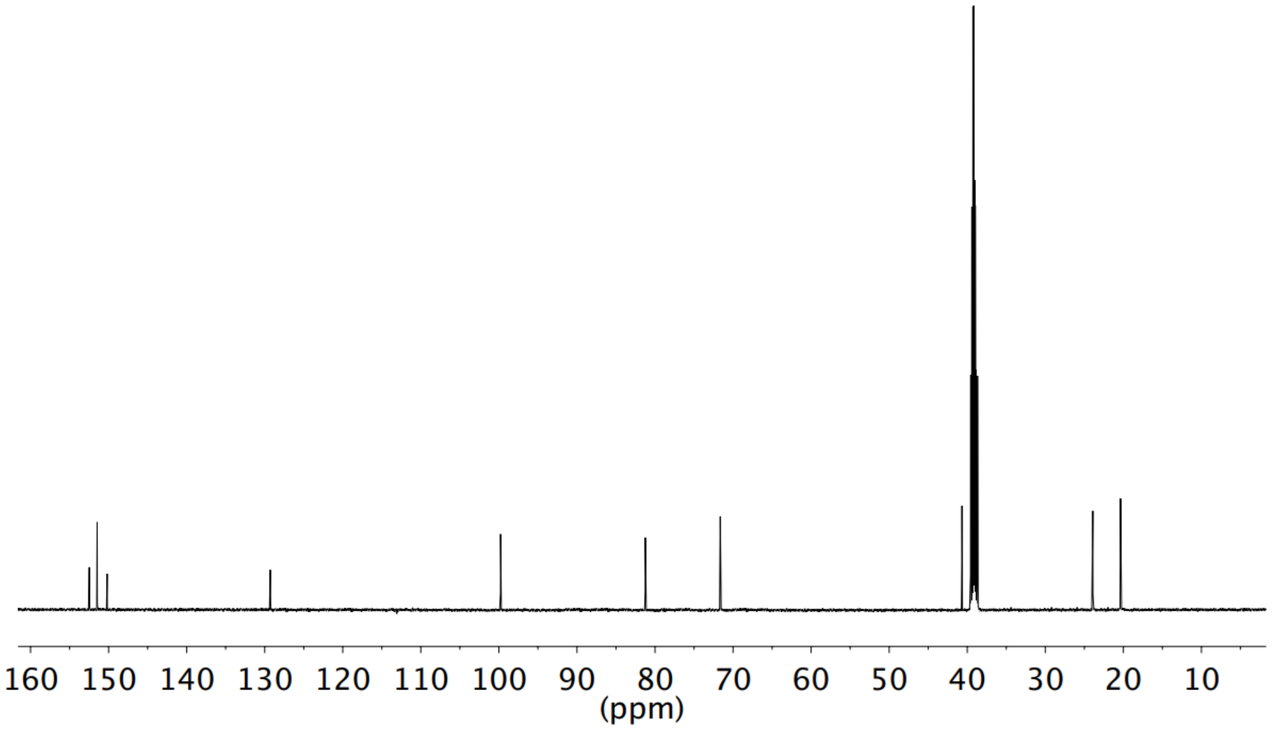

2.2 *Int-II*

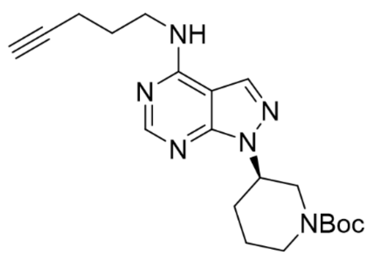
NH
N
N
N
N
NBoc

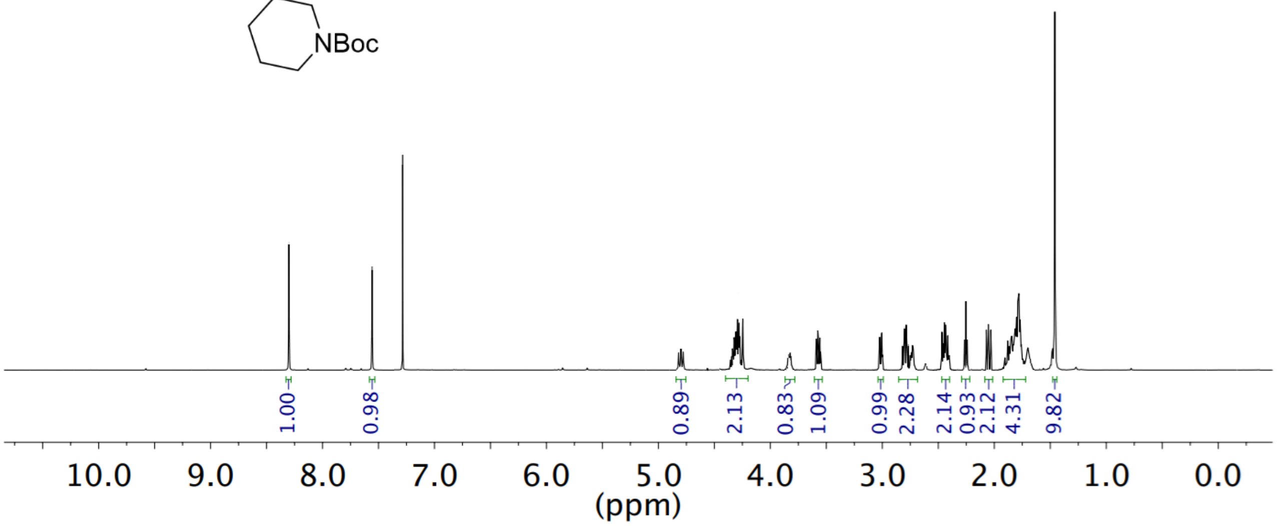
1.00
0.98
0.89
2.13
0.83
1.09
0.99
2.28
2.14
0.93
2.12
4.31
9.82
10.0
9.0
8.0
7.0
6.0
5.0
4.0
3.0
2.0
1.0
0.0
(ppm)

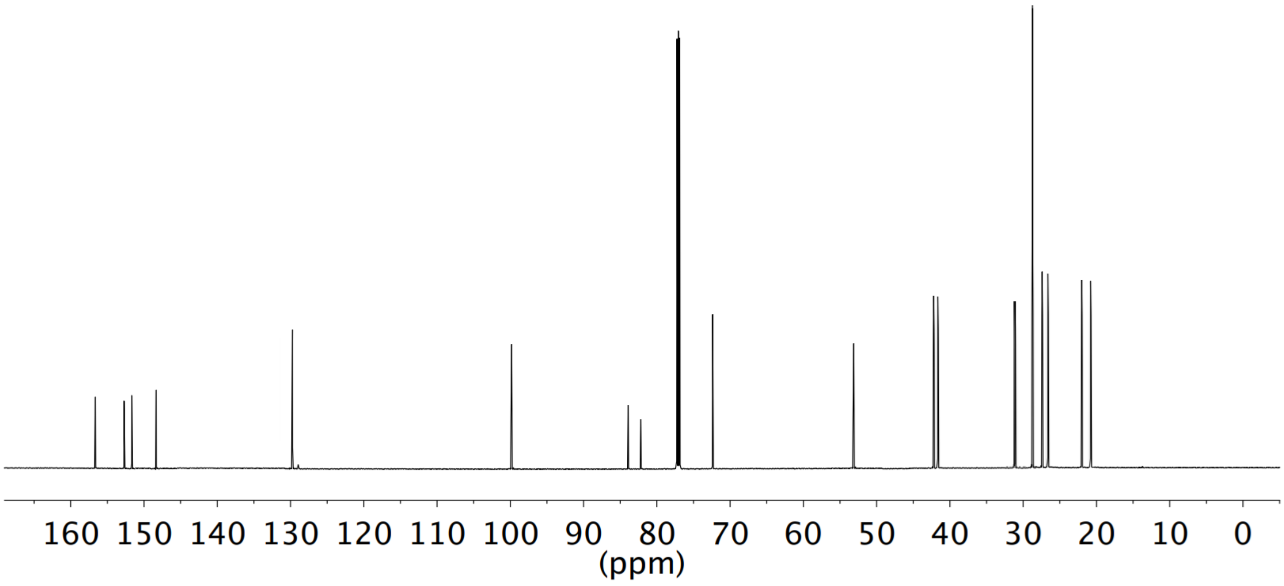
160
150
140
130
120
110
100
90
80
70
60
50
40
30
20
10
0
(ppm)

2.3 Probe 1

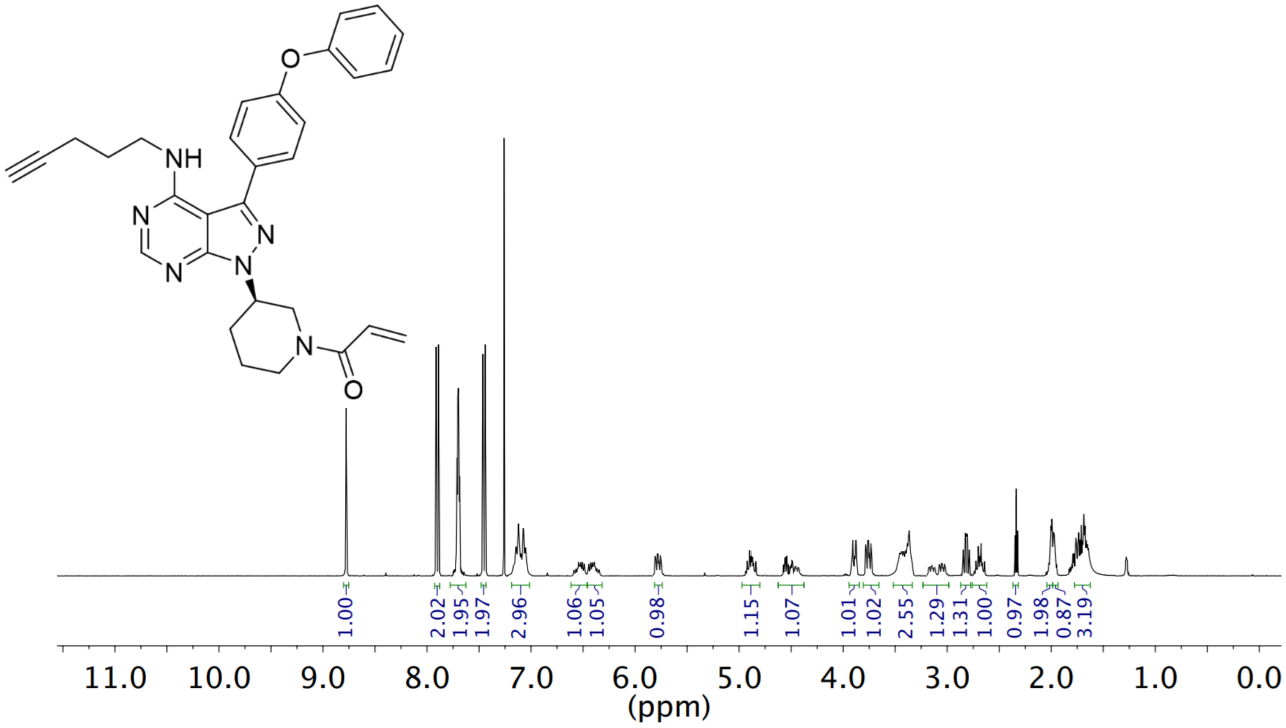

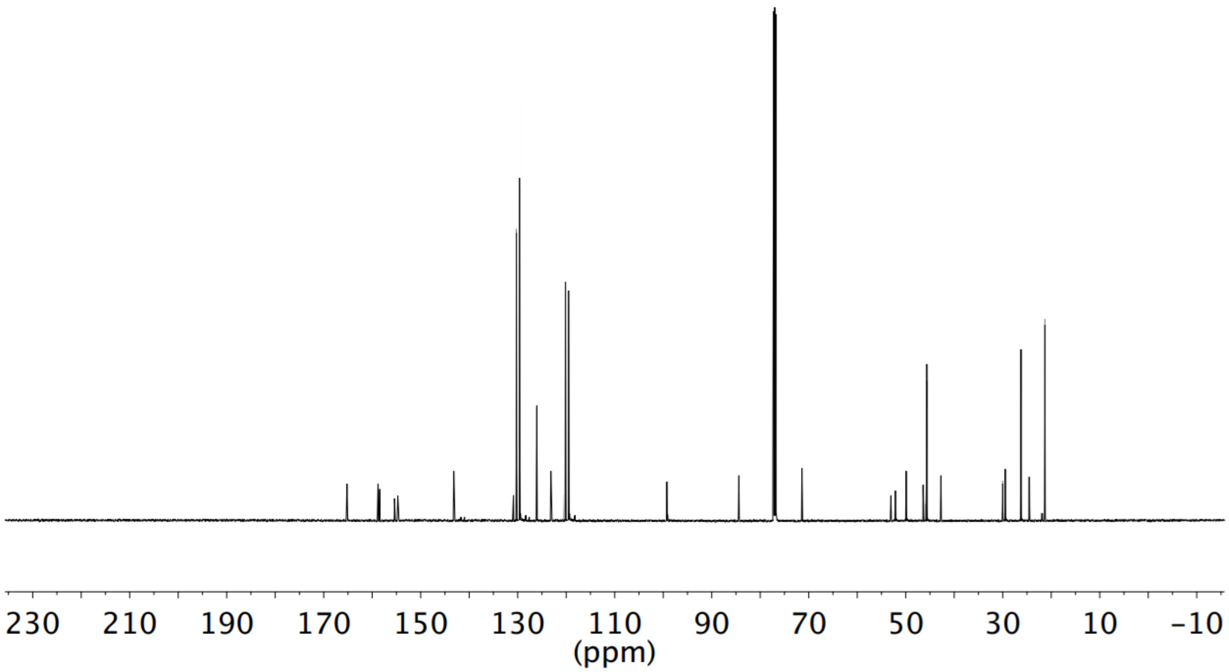

2.4 Probe 2

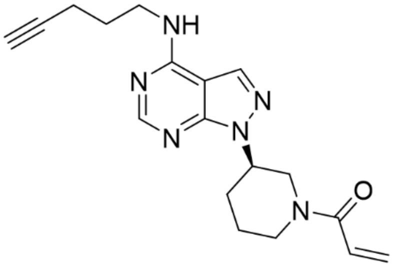
NH
N
N
N
N
N
O

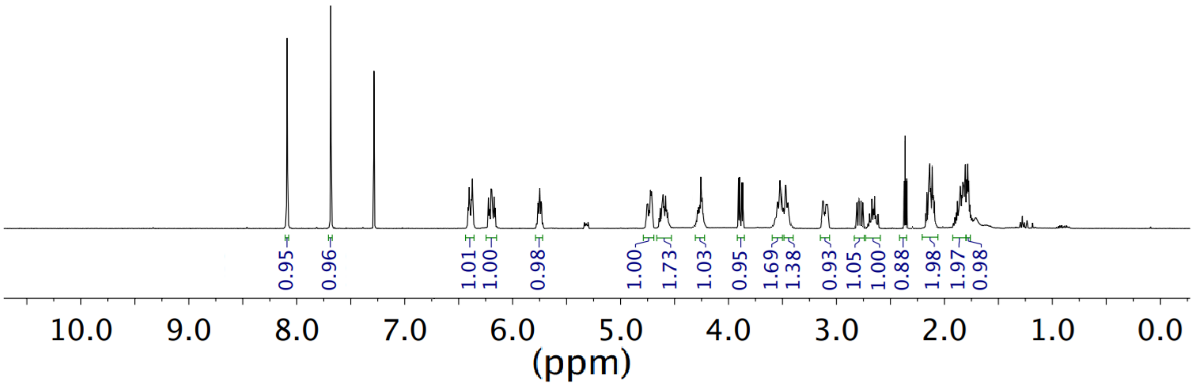
0.95
0.96
1.01
1.00
0.98
1.00
1.73
1.03
0.95
1.69
1.38
0.93
1.05
1.00
0.88
1.98
1.97
0.98
10.0 9.0 8.0 7.0 6.0 5.0 4.0 3.0 2.0 1.0 0.0
(ppm)

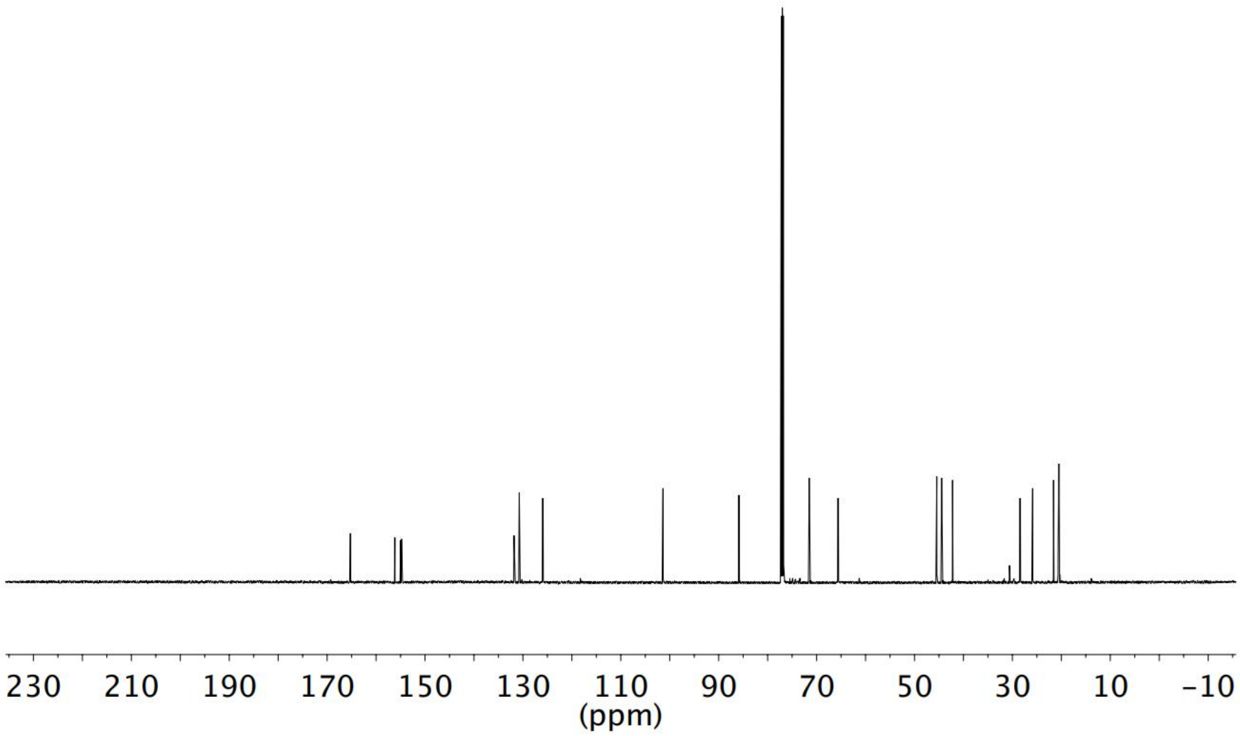
230 210 190 170 150 130 110 90 70 50 30 10 −10
(ppm)

2.5 Probe 3

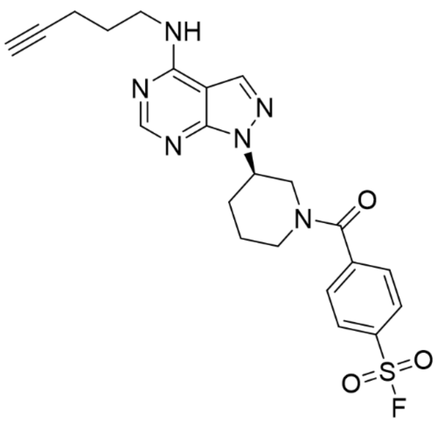
NH
N
N
N
N
O
O=S=O
F

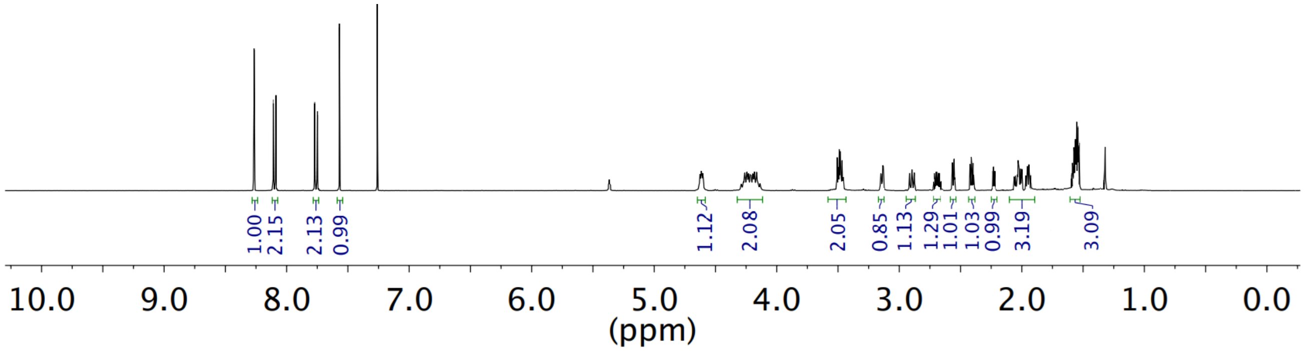
1.00
2.15
2.13
0.99
1.12
2.08
2.05
0.85
1.13
1.29
1.01
1.03
0.99
3.19
3.09
10.0 9.0 8.0 7.0 6.0 5.0 4.0 3.0 2.0 1.0 0.0
(ppm)

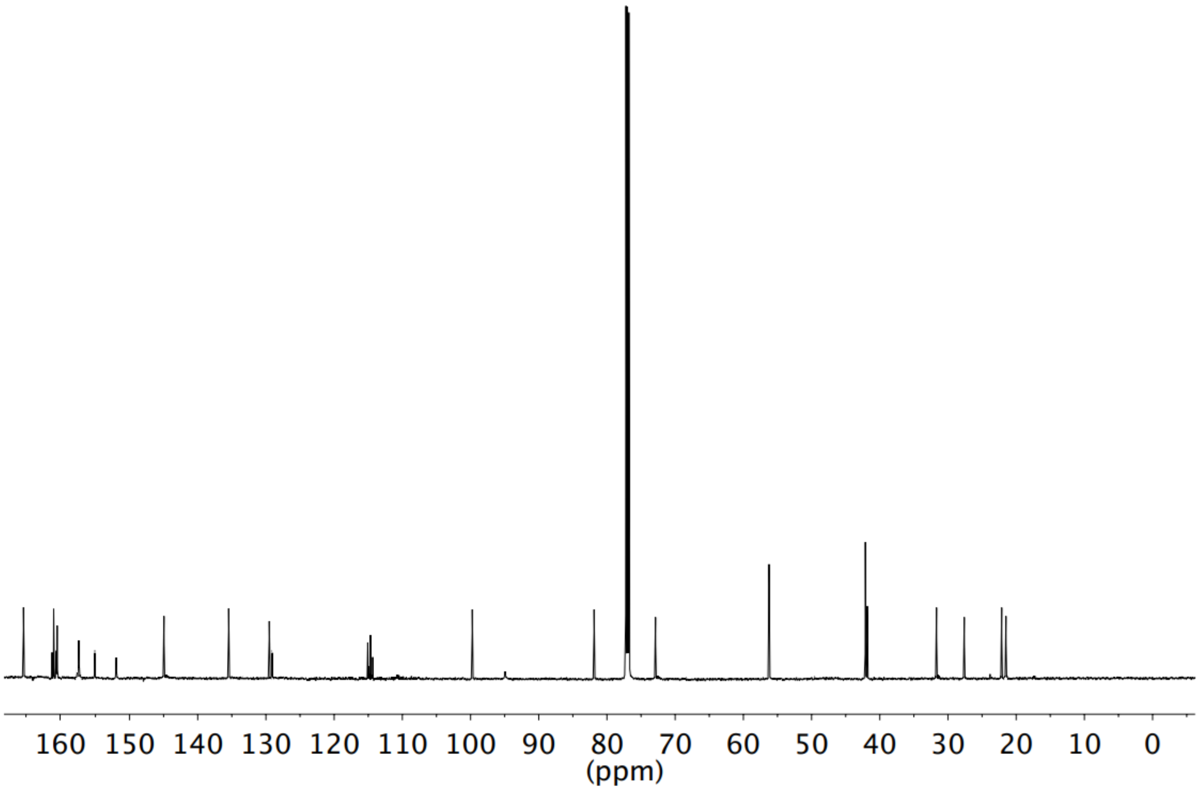
160 150 140 130 120 110 100 90 80 70 60 50 40 30 20 10 0
(ppm)

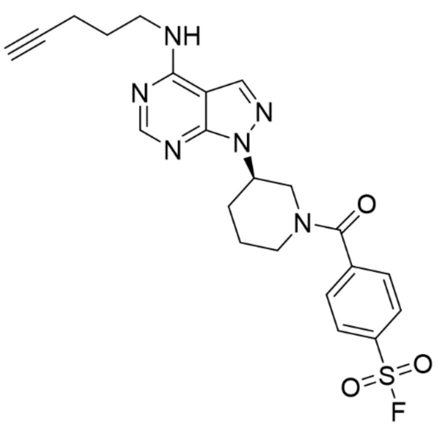
NH
N
N
N
N
O
O
S
O
F

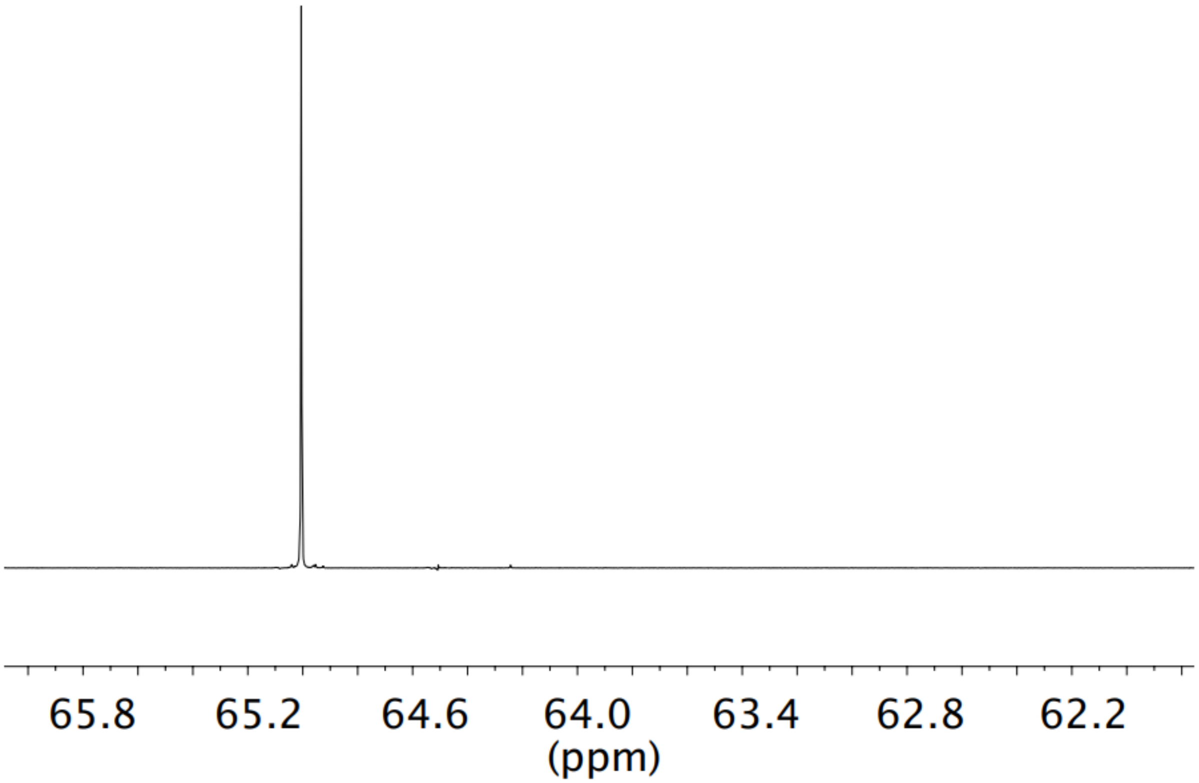
65.8
65.2
64.6
64.0
63.4
62.8
62.2
(ppm)
